# Discovery of Novel, Selective, and Nonbasic Agonists for the Kappa-Opioid Receptor Determined by Salvinorin A-Based Virtual Screening

**DOI:** 10.1021/acs.jmedchem.4c00590

**Published:** 2024-08-01

**Authors:** Kristina Puls, Aina-Leonor Olivé-Marti, Siriwat Hongnak, David Lamp, Mariana Spetea, Gerhard Wolber

**Affiliations:** † Department of Pharmaceutical Chemistry, Institute of Pharmacy, Freie Universität Berlin, Königin-Luise-Str. 2-4, 14195 Berlin, Germany; ‡ Department of Pharmaceutical Chemistry, Institute of Pharmacy and Center for Molecular Biosciences Innsbruck (CMBI), University of Innsbruck, Innrain 80-82, 6020 Innsbruck, Austria

## Abstract

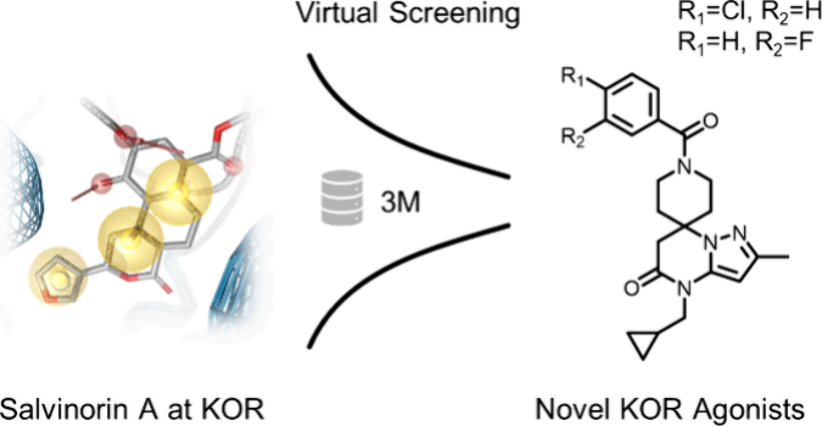

Modulating the kappa-opioid receptor (KOR) is a promising strategy for treating various human diseases. KOR agonists show potential for treating pain, pruritus, and epilepsy, while KOR antagonists show potential for treating depression, anxiety, and addiction. The diterpenoid Salvinorin A (SalA), a secondary metabolite of *Salvia divinorum*, is a potent and selective KOR agonist. Unlike typical opioids, SalA lacks a basic nitrogen, which encouraged us to search for nonbasic KOR ligands. Through structure-based virtual screening using 3D pharmacophore models based on the binding mode of SalA, we identified novel, nonbasic, potent, and selective KOR agonists. *In vitro* studies confirmed two virtual hits, **SalA-VS-07** and **SalA-VS-08**, as highly selective for the KOR and showing G protein-biased KOR agonist activity. Both KOR ligands share a novel spiro-moiety and a nonbasic scaffold. Our findings provide novel starting points for developing therapeutics aimed at treating pain and other conditions in which KOR is a central player.

## Introduction

Pain medications have been in use for a long time,^1^ but the safe and effective treatment of pain is still an area of unmet medical need. Approved opioids are mu-opioid receptor (MOR) agonists that provide strong analgesia. However, they also cause serious side effects, such as respiratory depression, constipation, sedation, analgesic tolerance, physical dependence, and addiction.^2,3^ Opioids are currently indispensable for the treatment of severe pain conditions.^2,4^ As a result of promiscuous opioid prescribing, the opioid crisis has emerged in the United States, with thousands of opioid-related deaths and hospitalizations each year.^5^ This crisis emphasizes the critical need for safer pain medications. In addition to the MOR, there are the kappa-opioid receptor (KOR), the delta-opioid receptor (DOR), and the nonclassical opioid receptor nociceptin/orphanin FQ peptide (NOP).^6^ All opioid receptor types belong to the family of G protein-coupled receptors (GPCRs) with seven transmembrane domains.^7^ Opioid receptor activation can provide analgesia but with different side effect profiles.^2,4,8^ The knowledge that activation of the KOR, opposite to the MOR, does not produce euphoria, respiratory depression, or risk of overdose^9^ has stimulated the interest in discovering drugs acting on the KOR as potential pain therapeutics.^10−16^ Overall, modulation of KOR signaling is a promising strategy for developing pharmacotherapies for several human diseases, by either activating (treatment of pain,^13,16^ pruritus,^16^ and epilepsy^17^) or blocking (treatment of depression,^13,18^ anxiety,^13,18^ and addiction^13^) the receptor. However, the generation of selective KOR agonists is challenging due to the strong binding site similarity of the opioid receptor subtypes.

**Figure 1:**
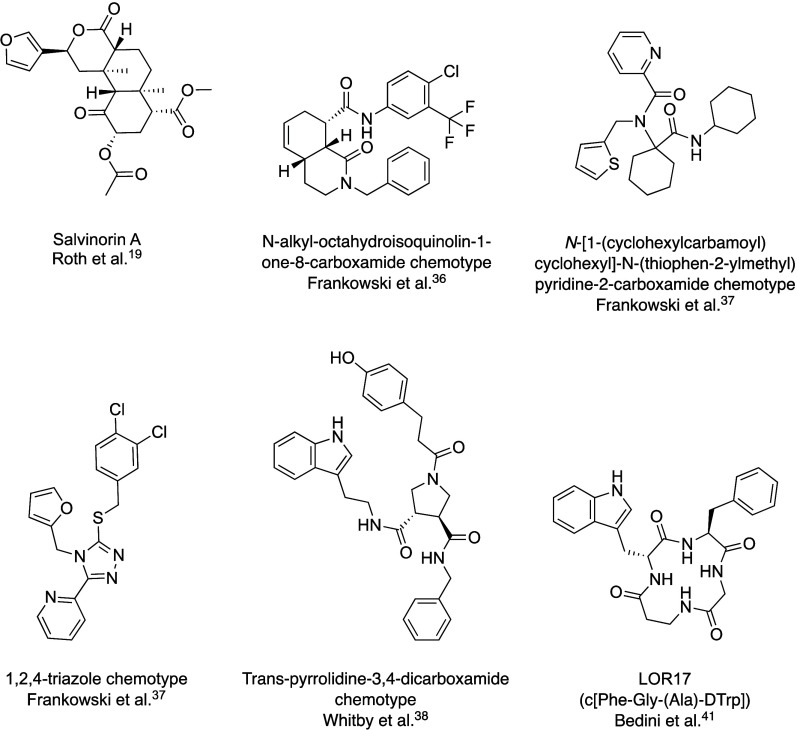
Nonbasic ligand scaffolds with reported activity at the KOR.

Salvinorin A (SalA) is a natural product (NP) of *Salvia divinorum*(19) (Figure 1). SalA was the first naturally occurring non-nitrogenous KOR agonist to be discovered,^19^ having high affinity and selectivity for the KOR.^19−22^ Notably, SalA lacks the basic amine found in prototypic opioids, which was previously believed to be essential for the interaction with opioid receptors.^23−26^ SalA was the first nonbasic opioid to be discovered, and much effort has been devoted to examining its exceptional KOR potency and selectivity profile. However, the lack of an active-state KOR crystal structure until 2018 (PDB-ID: 6B73(24)) and the numerous conflicting proposed binding modes of SalA at the KOR^20,24,27−33^ have hindered the elucidation of SalA’s selectivity determinants to the KOR. In addition, the potential clinical use of SalA is severely limited due to its unfavorable pharmacokinetics and strong hallucinogenic properties.^34,35^ However, the discovery of SalA has led to the idea of selectively targeting the KOR using nonbasic ligands.

Despite the pharmacological potential, the number of known basic KOR ligands surpasses the number of reported nonbasic ligands by far, with only a small number of nonbasic ligand scaffolds identified. Frankowski and co-workers^36^ reported a series of isoquinoline derivatives (*N*-alkyl-octahydroisoquinolin-1-one-8-carboxamides) as novel, potent, nonbasic, and KOR-selective ligands (Figure 1). This compound series showed good off-target selectivity against 38 nonopioid GPCRs. Two years later, Frankowski and co-workers^37^ published two new nonbasic KOR agonists obtained by a high throughput screening campaign, particularly the *N*-[1-(cyclohexylcarbamoyl)cyclohexyl]-*N*-(thiophen-2-ylmethyl)pyridine-2-carboxamide chemotype and the 1,2,4-triazol scaffold (Figure 1). In an attempt to identify small molecules mimicking β-turn conformations, Whitby and co-workers^38^ discovered the nonbasic trans-pyrrolidine-3,4-dicarboxamide scaffold (Figure 1). The fungal metabolite collybolide^39^ was thought to have KOR activity, but resynthesis by Shevick et al. recently showed that this compound was mischaracterized as a KOR agonist.^40^ Bedini and co-workers^41^ identified the cyclic tetrapeptide LOR17 (c[Phe-Gly-(Ala)-DTrp], Figure 1) to be a selective KOR agonist that displays favorable G protein bias with an improved safety profile compared to prototypical KOR agonists. To date, SalA remains the most extensively studied nonbasic scaffold. Approximately 600 derivatives of SalA have been synthesized and tested in KOR affinity or functional assays, albeit often with moderate or absent activity.^42−44^ Despite the extensive SAR around SalA, there is little information about the in vivo pharmacology of SalA derivatives. However, in a previous study,^40^ we have reviewed the extensive published experimental data around SalA and its derivatives and predicted a putative binding mode of SalA at the KOR.

Historically, NPs have made a major contribution to pharmacotherapy.^45^ Despite a decline in interest in NPs in the 1990s and the exploration of synthetic databases, NP-based drug development is experiencing a revival.^46^ Between January 1981 and September 2019, 1881 drugs have been approved by the United States Food and Drug Administration (FDA) or comparable organizations, of which about a quarter are unaltered NPs, defined mixtures of NPs, or (semisynthetic) derivatives of NPs.^47^ If only small molecules (*n* = 1394) are considered, the proportion rises to one-third.^47^ NPs exhibit some advantages over synthetic compounds but also bring some challenges.^46^ They typically differ from synthetic compounds by greater rigidity, more sp^3^ hybridized carbon and oxygen atoms but fewer nitrogen and halogen atoms, more chiral centers, greater molecular mass, larger hydrophilicity, and a higher number of hydrogen bond acceptors and donors.^46,47^ Overall, NPs cover a wider chemical space than synthetic compounds.^48^ In addition, NPs possess an enriched bioactivity due to evolutionary development.^46^ Frequent problems in NP-based drug discovery are labor-intensive synthesis or extraction processes, problems with the cultivation or extraction of sufficient product from the original source, or the complex elucidation of the molecular mechanisms behind effects in phenotypic screening assays.^46^ However, exploration of the chemical space around NPs is useful for the development of novel drug candidates. Thus, our putative KOR-SalA binding mode represents a valuable starting point for a virtual screening to search for new nonbasic opioids. Virtual screening has already been proven to be a suitable method for the discovery of novel GPCR ligands.^49−51^

In this study, we conducted a 3D pharmacophore-based virtual screening campaign aiming to discover novel, nonbasic, and selective KOR agonists as potential therapeutics. We employed our recently published binding mode of SalA at the KOR^52^ as a starting point for the 3D pharmacophore generation. Selected virtual screening hits were subjected to pharmacological *in vitro* evaluation resulting in the discovery of two new nonbasic KOR agonists with nanomolar affinity and potency, as well as very good selectivity for the KOR over the other opioid receptor subtypes, MOR and DOR. We identified **SalA-VS-07** as a partial agonist at the KOR, while **SalA-VS-08** shows a full agonist profile at the KOR with both compounds having a G protein-biased agonist profile. Both hit compounds are NP mimicking compounds that share a novel scaffold containing a spiro moiety.

## Results

### SalA-Based Virtual Screening: Identification of Novel NP Derived KOR Ligands

**Figure 2:**
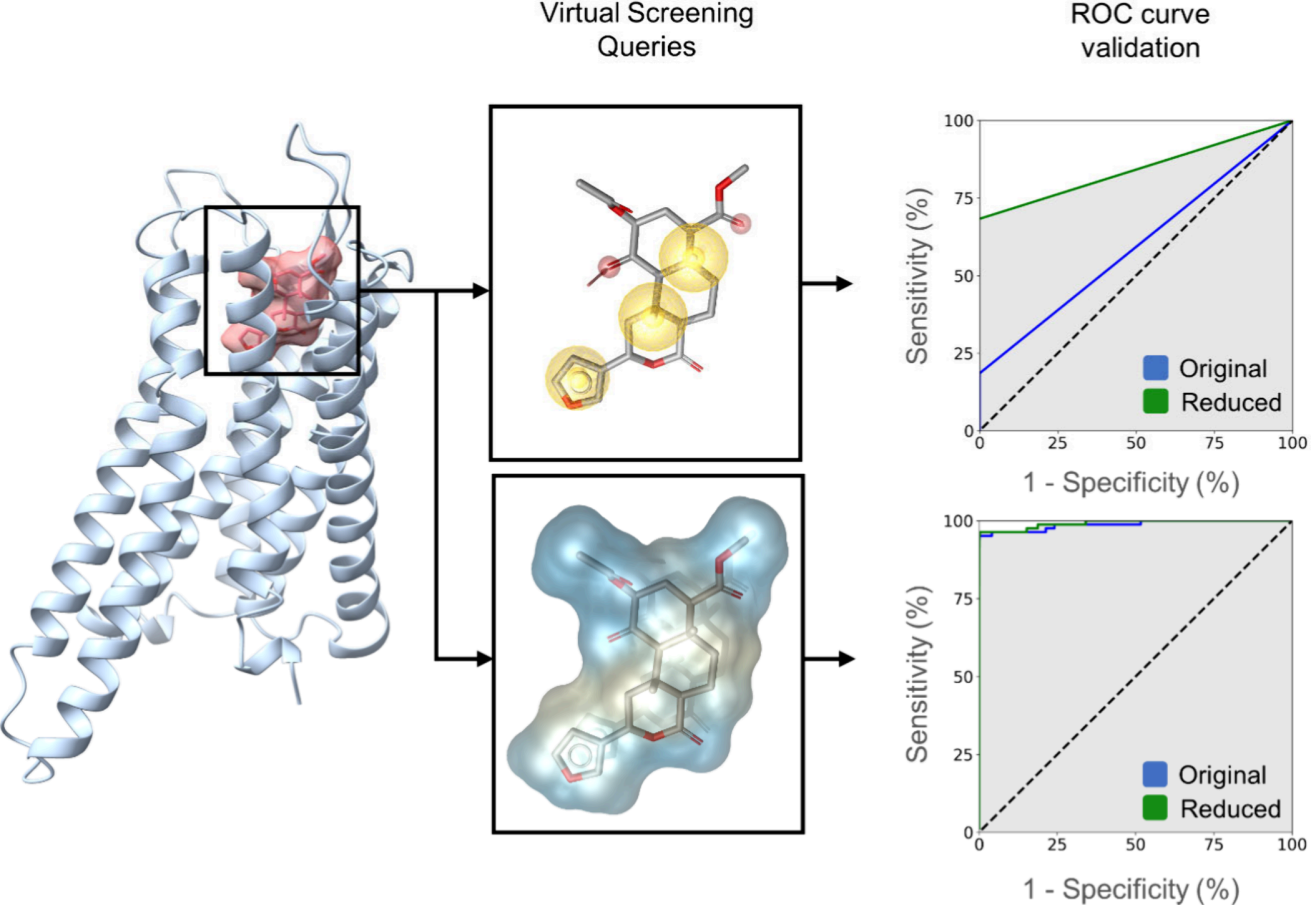
Virtual screening queries and validation. Previously published binding mode^52^ of SalA at the KOR used for query generation is shown on the left side. At the top, the 3D pharmacophore information used as query for the screening in LigandScout^53,100^ is depicted. Exclusion volume spheres are not shown for the sake of clarity. At the bottom, the shape information used as query for the ROCS^101,102^ screening is shown. ROCS additionally takes 3D pharmacophore features into account. On the right sight, the respective receiver operating characteristic (ROC) curves are shown. The queries derived from the original 3D pharmacophore of the KOR-SalA complex (blue) are compared to the reduced 3D pharmacophore queries missing the SalA-C2 HBA feature (green). The dashed black line on both sides indicates a random line. Gray area shows the area under the curve for the reduced 3D pharmacophore query.

We used structure-based virtual screening to search for nonbasic KOR ligands. Two virtual screening workflows were implemented using LigandScout^53,54^ (Inte:Ligand, Vienna, Austria) and ROCS (OpenEye/Cadence Molecular Sciences, Santa Fe, NM). The first workflow was based on structure-based 3D pharmacophores developed with LigandScout, while the second workflow was based on three-dimensional shape and chemical feature similarity using ROCS. Due to the absence of an experimentally determined structure of the SalA-KOR complex at the start of the study, we utilized our previously published binding hypothesis of SalA at the KOR^52^ to generate both of our virtual screening queries (Figure 2). Further details about the query generation can be found in the Experimental Section.

Before conducting the virtual screening campaign, we validated and fine-tuned the SalA 3D pharmacophore query using receiver operating characteristic (ROC) curves^55^ to ensure optimal performance in virtual screening. A set of 82 nonbasic KOR agonists, obtained from the ChEMBL database,^56^ was used as active compounds. A number of 4100 decoys were generated using DUD-E^57^ yielding in a ratio of actives to decoys of 1:50. The data set only includes nonbasic KOR agonists, primarily SalA analogues, due to the limited number of different nonbasic KOR ligand scaffolds known. Basic agonists were excluded because a similar binding mode of basic and nonbasic KOR ligands cannot be assumed. Several mutational studies suggest that residues above the morphinan binding site are crucial for SalA, including Y312^7.35^, Y313^7.36^, and Y119^2.64^ as well as extracellular loop 2.^20,27,32^ Our previously published binding mode of SalA at the KOR suggests a distinct SalA binding site as well.^52^ Additional information regarding the database curation process can be found in the Experimental Section. The initial 3D pharmacophore of SalA bound to the KOR comprises three hydrophobic contact features (HY) and three hydrogen bond acceptor features (HBA).^52^ ROC curve evaluation indicated that the omission of the SalA-C2 HBA yields better virtual screening performance, in particular for the virtual screening workflow performed in LigandScout (Figure 2). Both 3D pharmacophores in the ROCS screening perform similarly. However, we omitted the SalA-C2 HBA feature, which slightly improved the early enrichment. For the sake of conformity, we performed virtual screening with the reduced pharmacophore for both methods. The optimized queries yielded the following results: The LigandScout 3D pharmacophore screening achieved a sensitivity of 68% (56 out of 82 actives), with a total of 67 hits (11 false positives, FP). The area under the curve (AUC) is 0.85. In the ROCS screening, 79 out of the 82 active compounds were ranked within the top 87 molecules with the first decoy at position 59, followed by positions 67, 74, 79, and 83–86. The remaining three active compounds were ranked at positions 702, 848, and 1476. The AUC is 0.99.

The screened molecule libraries include both NP libraries and synthetic compound libraries (Table 1). NPs typically contain fewer nitrogen atoms than synthetic compounds, and their library size is much smaller. The screened NP or NP-derived databases were Analyticon’s NATx and MEGx libraries,^58^ as well as Selleckchem’s NP library.^59^ The Enamine database, which comprises Enamine’s advanced, functional, HTS, and premium collections, was screened for synthetic compounds.^60^ Both virtual screening methods were used in parallel to screen a total of around 3 million molecules.

**Table 1:** Composition of the Screened Compound Databases

natural product library	synthetic compound library	number of molecules	percent
NATx (Analyticon)		33,717	1.44%
MEGx (Analyticon)		6,541	
Natural product library (Selleckchem)		2,724	
	Advanced Collection (Enamine)	640,219	98.46%
	Functional Collection (Enamine)	119,088	
	HTS Collection (Enamine)	2,146,100	
	Premium Collection (Enamine)	41,281	
		Total: 2,989,670	100%

**Figure 3:**
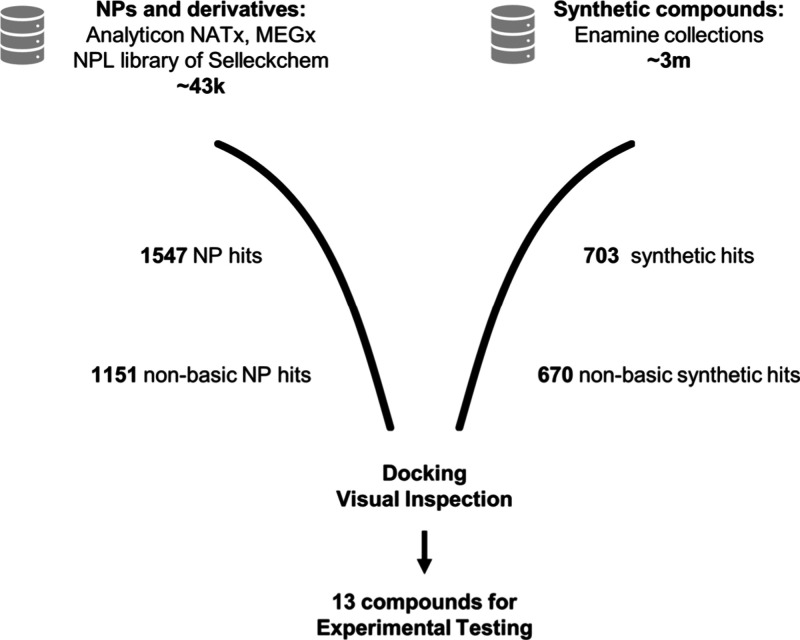
Schematic workflow of the virtual screening campaign, including the number of data points per step. Hits were summarized for both virtual screening methods. By default, ROCS retrieves the 500 best fitting compounds. Enamine databases screened were advanced, functional, HTS, and premium collections. NPL = Natural product library of Selleckchem.

**Figure 4:**
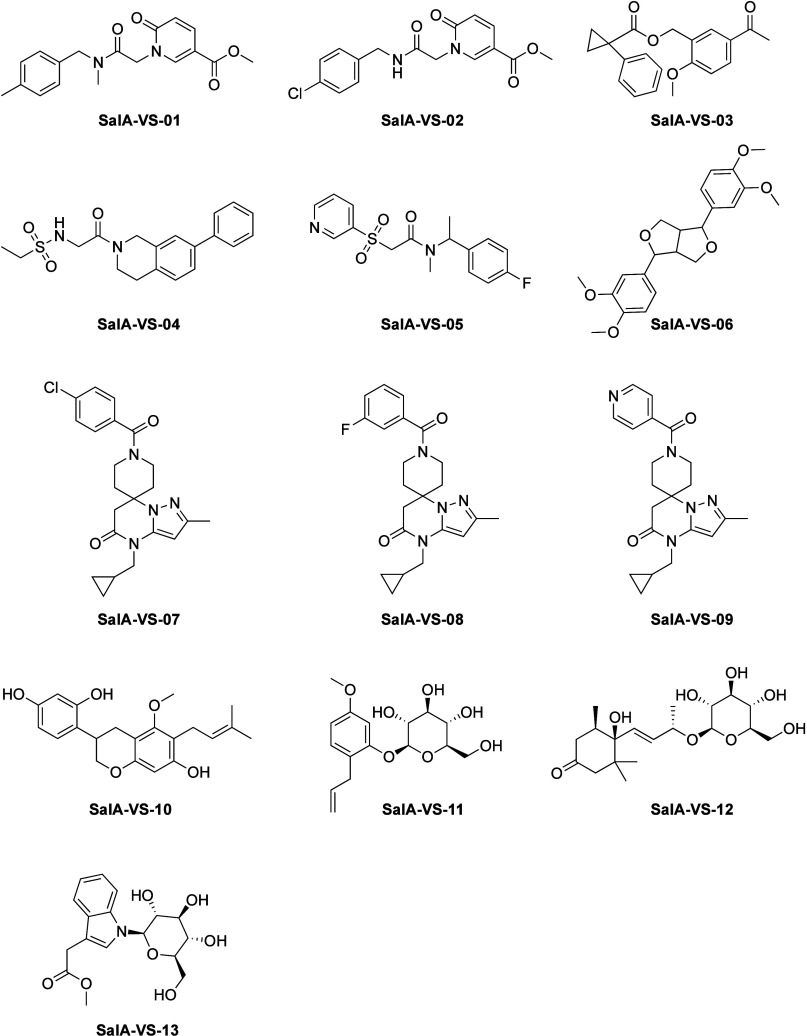
Chemical structures of virtual hits selected for experimental testing.

The virtual screening workflow is illustrated in Figure 3. The LigandScout screening resulted in 250 hits, while the ROCS screening resulted in 2000 hits. The hit lists were combined, and charged ligands were filtered out using RDKit v2022.03.3^61^ and KNIME v4.5.2,^62^ resulting in 1821 nonbasic hits. These hits were then docked into the active-state KOR X-ray crystal structure (PDB-ID: 6B73(24)) to generate plausible binding modes. To evaluate the initial docking, we calculated the 3D pharmacophore score in LigandScout toward the starting binding mode of SalA. The docking poses were scored based on their ability to fulfill the query pharmacophore features, and only those with a score greater than zero were kept for further evaluation. We then visually inspected the remaining docking poses and carefully selected the best fitting molecules for the experimental testing. The manual selection criteria primarily include high 3D pharmacophore scores and high Gaussian shape similarity scores^63,64^ toward SalA. Additionally, good physicochemical properties of the hit compounds were considered, such as a low number of rotatable bonds (≤10, with one exception) and a molecular weight of ≤500 Da. Criteria from our previous SalA investigation^52^ were also included, assuming similar binding modes for our nonbasic hit compounds compared to the nonbasic starting ligand SalA. Docking poses that interacted with residues highlighted as crucial for SalA affinity and potency in mutagenesis studies (Y312^7.35^, Y313^7.36^, V118^2.63^, Y139^3.33^, Q115^2.60^)^3,20,21,27,32^ were prioritized. We selected docking poses that align with the SAR data of SalA, particularly those that have an HBA at the position of SalA’s C2-acetoxy carbonyl group. This HBA is crucial for SalA’s potency to the KOR, as its primary metabolite Salvinorin B (SalB), which has a hydroxy group at its C2 position, is inactive.^34,35^ We further validated experimentally the 15 best molecules selected (Figure 4). It is worth noting that 14 out of the 15 hit molecules were obtained through ROCS screening, with 6 molecules derived from the synthetic Enamine database and 9 molecules, from NP libraries. This indicates that our developed 3D interaction model shows a clear preference for NP or NP derivatives.

### Pharmacological Evaluation: Identification of SalA-VS-07 and SalA-VS-08, Two NP Mimicking Compounds, as Novel, Selective, and G Protein-Biased KOR Agonists

**Figure 5:**
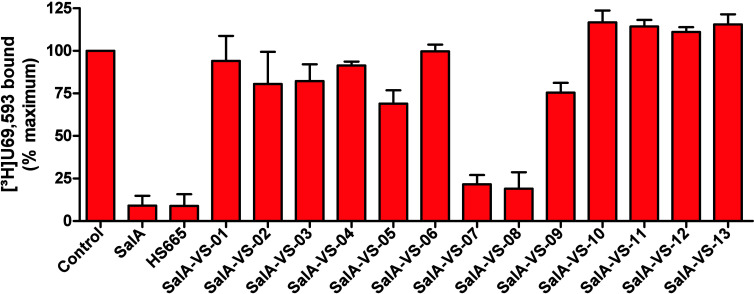
Screening of SalA-VS compounds for binding at the human KOR. Competitive inhibition of [^3^H]U69,593 binding by SalA-VS compounds and reference KOR ligands SalA and HS665 at the human KOR was measured in radioligand binding assays. Membranes from CHO cells stably expressing the human KOR were incubated with [^3^H]U69,593 in the absence (control) or presence of test compounds (10 μM). Values represent the mean ± SEM (*n* = 3).

**Figure 6:**
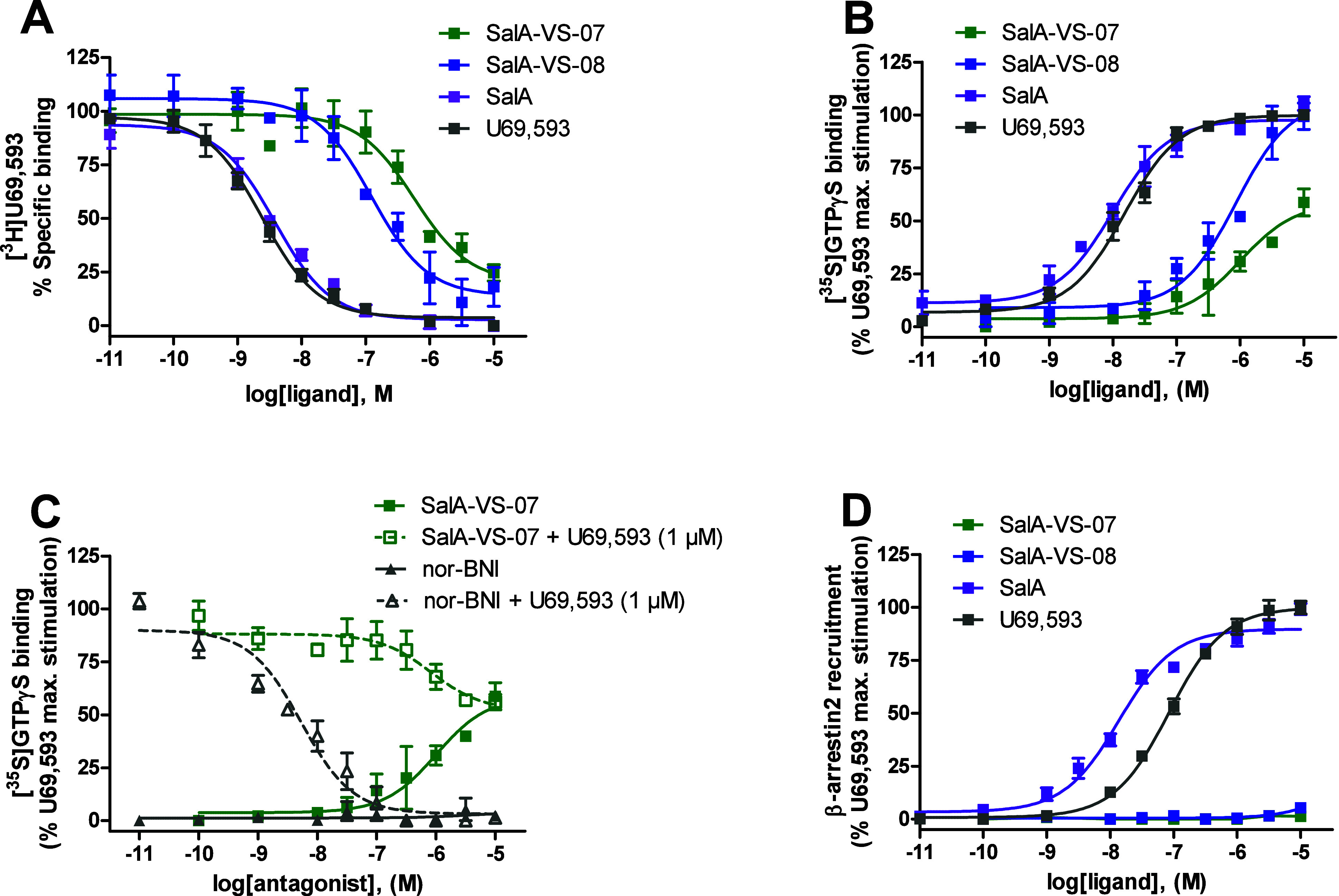
*In vitro* activity profiles of **SalA-VS-07** and **SalA-VS-08** at the human KOR. (A) Concentration-dependent inhibition by **SalA-VS-07**, **SalA-VS-08**, SalA, and U69,593 of [^3^H]U69,593 binding to membranes from CHO-hKOR cells determined in the radioligand competitive binding assay. (B) Concentration-dependent stimulation of [^35^S]GTPγS binding by **SalA-VS-07**, **SalA-VS-08**, SalA, and U69,593 in the [^35^S]GTPγS binding assay using membranes from CHO-hKOR cells. (C) Partial agonist activity at the human KOR of **SalA-VS-07**. [^35^S]GTPγS binding was measured in CHO-hKOR cell membranes incubated with increasing concentrations of **SalA-VS-07** or nor-BNI in the presence or in the absence of U69,593 (1 μM). (D) β-Arrestin2 recruitment activities of **SalA-VS-07**, **SalA-VS-08**, SalA, and U69,593 at the human KOR expressed in U2OS−β-arrestin2 cells were determined in the PathHunter β-arrestin2 assay. [^35^S]GTPγS binding and β-arrestin2 recruitment data are presented as the percentage of stimulation relative to the maximum effect of the reference KOR agonist U69,593. Values represent the mean ± SEM (*n* = 3–4).

**Figure 7:**
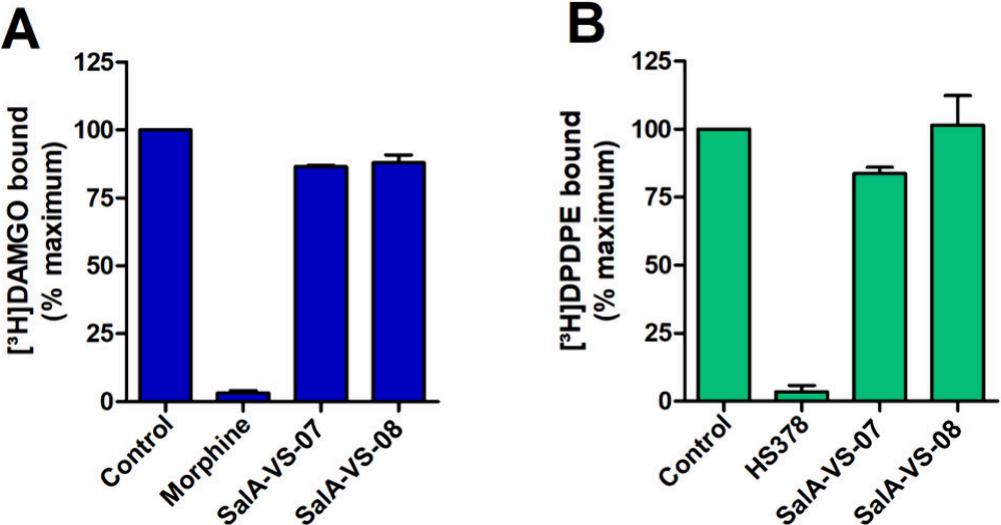
Binding of **SalA-VS-07** and **SalA-VS-08** at the human MOR and DOR determined by radioligand binding assays. Membranes from CHO cells stably expressing the human MOR or DOR were incubated with (A) the specific MOR radioligand [^3^H]DAMGO or (B) the specific DOR radioligand [^3^H]DPDPE in the absence (control) or presence (10 μM) of test compounds. Morphine (10 μM) and HS378 (10 μM) were used as the reference MOR and DOR ligands, respectively. Values represent the mean ± SEM (*n* = 3).

In the quest for the identification of novel, selective ligands at the KOR, the seven NP mimicking compounds, **SalA-VS-07** to **SalA-VS-13**, selected from the virtual screening campaign, were provided by AnalytiCon for experimental testing. The initial biological screening was performed using a competitive radioligand binding assay at the human KOR. The ability of the test compounds and the reference KOR ligands, SalA^19^ and HS665^65^ (Figure S1), all tested at 10 μM, to inhibit binding of the specific KOR radioligand [^3^H]U69,593 (Figure S1) was assessed with membranes from Chinese hamster ovary (CHO) cells stably expressing the human KOR (CHO-hKOR), according to the described procedure.^66^ Among the tested compounds, **SalA-VS-07** and **SalA-VS-08** inhibited [^3^H]U69,593 binding at the KOR by >50% (Figure 5). Therefore, they were selected for further investigations of their *in vitro* KOR activities. Both compounds produced concentration-dependent inhibition of [^3^H]U69,593 binding (Figure 6A), displaying good binding affinities (defined by the *K*_i_ value) in the nanomolar range at the human KOR (Table 2). **SalA-VS-08** showed about 6-fold increased affinity at the KOR than **SalA-VS-07** but with reduced binding affinities than the natural KOR ligand, SalA (Table 2). SalA, as expected, exhibited a high binding affinity, in the low nanomolar range, at the human KOR (*K*_i_ = 2.66 nM). To assess the selectivity of **SalA-VS-07** and **SalA-VS-08** for the KOR, competitive radioligand binding studies were performed with membranes from CHO cells stably expressing the human MOR or DOR, and using [^3^H]DAMGO and [^3^H]DPDPE (Figure S1), as specific MOR and DOR radioligands, respectively. As shown in Figure 7, **SalA-VS-07** and **SalA-VS-08** showed no substantial binding at the MOR and DOR at a concentration of 10 μM. In the same assays, the reference MOR and DOR ligands, morphine and HS378^67^ (Figure S1), respectively, showed significant binding at the specific opioid receptors (Figure 7).

**Table 2:** Binding Affinities and Functional Activities of **SalA-VS-07** and **SalA-VS-08** at the Human KOR^a^

	binding affinity^b^	G protein activation^c^		β-arrestin2 recruitment^d^	
Ligand	*K*_i_ (nM)	EC_50_ (nM)	*E*_max_ (%)	EC_50_ (nM)	*E*_max_ (%)
**SalA-VS-07**	423 ± 97	181 ± 49	48 ± 7	–e	
**SalA-VS-08**	68.5 ± 5.5	854 ± 60	108 ± 8	–e	
SalA	2.66 ± 0.75	11.8 ± 3.5	100 ± 1	14.5 ± 2.8	90.1 ± 1.0
U69,593	1.56 ± 0.34	16.3 ± 3.5	100	85.0 ± 5.7	100

a *E*_max_ (%) values represent the percentage relative to the maximal effect of U69,593 (as 100%). Values represent the mean ± SEM (*n* = 3–4).

b Determined in the radioligand competitive binding assays using membranes from CHO cells stably expressing the human KOR.

c Determined in the [^35^S]GTPγS binding assays using membranes from CHO cells stably expressing the human KOR.

d Determined in the PathHunter β-arrestin2 recruitment assay with U2OS cells coexpressing the hKOR and the enzyme acceptor tagged β-arrestin2 fusion protein.

e Denotes no measurable activity up to 10 μM.

Next, we have evaluated the *in vitro* functional activity of **SalA-VS-07** and **SalA-VS-08** at the human KOR in the guanosine-5′-*O*-(3-[^35^S]thio)-triphosphate ([^35^S]GTPγS) binding assay with CHO-hKOR cell membranes as described previously.^66^ Their *in vitro* G protein signaling at the KOR was compared to the reference KOR agonists, SalA and U69,593 (Figure 6B). Agonist potencies (EC_50_, nM) and efficacies (*E*_max_, %) to induce MOR-mediated G protein activation are listed in Table 2. Both **SalA-VS-07** and **SalA-VS-08** produced a concentration-dependent increase in the [^35^S]GTPγS binding in CHO-hKOR cell membranes although with distinct activation profiles. Whereas **SalA-VS-08** showed full efficacy (108% of U69,593) in inducing KOR-mediated G-protein activation, **SalA-VS-07** displayed partial agonism based on reduced efficacy (48% of U69,593). Notable is also the observation on the 5-fold lower agonist potency at the KOR of **SalA-VS-08** compared to **SalA-VS-07**. Experimentally, we established that **SalA-VS-07** displays a more potent KOR activation despite a lower binding affinity compared to **SalA-VS-08** (Table 2). However, **SalA-VS-07** and **SalA-VS-08** showed reduced agonist potency at the KOR in the [^35^S]GTPγS binding assay compared to SalA (Table 2). The functional profile of **SalA-VS-07** as a partial agonist was further confirmed by its partial inhibition of U69,593-induced stimulation of [^35^S]GTPγS binding (IC_50_ = 1274 ± 334 nM; Inhibition = 52 ± 4%), as compared to the full antagonism produced by the selective KOR antagonist, nor-binaltorphimine (nor-BNI, Figure S1) (IC_50_ = 1.80 ± 0.71 nM; Inhibition = 92 ± 6%) (Figure 6C).

In addition to G protein activation, another important signaling event following KOR stimulation is agonist-induced β-arrestin2 recruitment. In this context, β-arrestin2 has been linked to severe adverse effects, thereby highlighting the potential of G protein-biased KOR ligands to design therapeutics with improved side effect profile.^9,68,69^ We next explored the functional properties of **Sal-VS-07** and **Sal-VS-08** to recruit β-arrestin2 at the KOR in the PathHunter β-arrestin2 recruitment assay using U2OS cells coexpressing the human KOR and the enzyme acceptor tagged β-arrestin2 fusion protein. Remarkably, **Sal-VS-07** and **Sal-VS-08** failed to induce β-arrestin2 recruitment upon activation of the KOR, whereas the reference KOR ligands, SalA and U69,593, full agonists of β-arrestin2 with EC_50_ values of 14.5 and 85 nM, respectively, effectively recruited β-arrestin2 (Figure 6D, Table 2). Since both compounds exhibit significant efficacy at the KOR for G protein activation in the [^35^S]GTPγS binding assay (Figure 6B), there is an evident bias in favor of G protein signaling. β-Arrestin2 recruitment was too low in the range of tested concentrations for **Sal-VS-07** and **Sal-VS-08** to permit a formal determination of a bias factor.

Both **SalA-VS-07** and **SalA-VS-08** as well as the inactive **SalA-VS-09** (Figure 4) share the same common scaffold only differing at the aromatic substitution at the benzamide moiety. **SalA-VS-07** contains a 4-chlorobenzamide, **SalA-VS-08**, a 3-fluorobenzamide, and **SalA-VS-09**, a pyridine-4-carboxamide structure. The corresponding experimental data provide first insights into the SAR of our newly developed 2-methylspiro[6*H*-pyrazolo[1,5-*a*]pyrimidine-7,4′-piperidine]-5-one scaffold series. The introduction of a halogen moiety in para or meta position of the benzamide substructure facilitates KOR activity as its absence in **SalA-VS-09** represents an interesting activity cliff.

## Discussion

Based on our recently reported binding hypothesis of SalA at the KOR,^52^ 3D pharmacophore-based virtual screening discovered two NP mimicking compounds as novel, selective, and nonbasic KOR agonists. This is the first time that new nonbasic opioids have been rationally rather than serendipitously discovered. These new KOR agonists, **SalA-VS-07** and **SalA-VS-08**, share the 2-methylspiro[6*H*-pyrazolo[1,5-*a*]pyrimidine-7,4′-piperidine]-5-one scaffold (Figure 4). Both **SalA-VS-07** and **SalA-VS-08** were highly selective for the KOR and showed G protein-biased KOR agonist activity *in vitro*.

To assess the novelty of our new spiro moiety-containing scaffold (Figure 4), we conducted a thorough literature review. A few studies have already reported on spiro moiety-containing opioids.^70−82^ However, these compounds share only limited similarities to **SalA-VS-07** and **SalA-VS-08**. Moreover, the previously reported compounds were primarily developed for the NOP receptor^70,71,75−79,81,82^ and DOR^73,74^ and often do not report activity values for the KOR.^71,77,80^**SalA-VS-07** and **SalA-VS-08** contain a spiro-piperidine substructure. Spiro-piperidine opioids have already been reported for the DOR and NOP receptor.^70,71,73,74,76,79,81,82^ However, unlike **SalA-VS-07** and **SalA-VS-08**, many of the spiro compounds found in the literature possess a spiro group of a 6-membered ring connected to a 5-membered ring^70,71,76,79,81,82^ or pyrans or oxidized pyrans.^72−74,77,78^ Only Mustazza and co-workers reported a spiro moiety encompassing two nitrogen containing 6-membered rings similar to our discovered scaffold.^75^ Despite this similarity, the molecules contain a quinazoline substructure dissimilar to our hit compounds.^75^ The structure–activity relationship (SAR) data provided by Mustazza and co-workers indicate all compounds far less potent at the KOR (micromolar range) compared to our newly discovered lead compounds (nanomolar range). Additionally, most of their compounds are less KOR selective. Unlike **SalA-VS-07** and **SalA-VS-08**, all spiro opioids found in the literature contain a basic amine. From the results of our literature review, we concluded that our spiro moiety-containing scaffold is indeed novel for opioid receptor modulation.

In this study, we explored a nonconventional chemical space for opioid receptor ligands due to the nonbasic nature of the compounds screened. The focus on nonbasic ligands increased the risk of the virtual screening campaign but also increased the novelty of experimentally confirmed hits. We investigated the chemical space covered by our 1821 virtual screening hits using Principal component analysis (PCA^83^) and Uniform Manifold Approximation and Projection (UMAP^84^) via ChemPlot^85^ (Figures S2 and S3). The analyses show that our selected hits for the experimental testing cover a large space within the chemical space of all virtual screening hits.

Despite the much larger size of the synthetic libraries screened, only five (**SalA-VS-01**–**SalA-VS-05**) of the 13 substances for experimental validation were derived from the synthetic Enamine databases. The remaining eight substances belong to NP or NP-derived libraries. In particular, **SalA-VS-06** originates from Selleckchem’s NPL, **SalA-VS-10**–**SalA-VS-13**, from Analyticon’s MEGx, and **SalA-VS-07**–**SalA-VS-09**, from Analyticon’s NATx. The NATx library contains NP-inspired chemical compounds. Thus, lead structures **SalA-VS-07** and **SalA-VS-08** represent NP mimicking compounds. In particular, **SalA-VS-07** and **SalA-VS-08** resemble spiroaminals and spiroacetals, albeit with some chemical modifications. Spiroaminals and spiroacetals are ubiquitous in NPs and can be found for example in polyketides.^86−91^

There is a long history of modulation of the opioid receptor system using NPs. Besides the well-known morphine,^45,92^ its naturally occurring derivatives like codeine,^93^ and already discussed SalA, kratom alkaloids such as mitragynine, 7-OH mitragynine, and mitragynine pseudoindoxyl modulate MOR and KOR^94^ (Figure S4). Further NP-opioids are the naturally occurring peptides rubiscolin-5 (YPLDL) and rubiscolin-6 (YPLDLF)^95^ (Figure S4). Additionally, opioid receptors can be modified by endogenous opioid peptides.^96^ Our lead compounds **SalA-VS-07** and **SalA-VS-08** represent a novel chemical category within natural compounds demonstrated to be active at the KOR.

**SalA-VS-07** and **SalA-VS-08** are nonbasic KOR ligands with a G protein biased agonist profile at the KOR but with different efficacies in inducing activation of the KOR. While **SalA-VS-08** shows a full agonistic profile in the G protein activation assay compared to the prototypical KOR agonist U69,593, **SalA-VS-07** exhibits partial agonistic properties at the KOR (Figure 6B). Furthermore, **SalA-VS-07** and **SalA-VS-08** were identified as G protein-biased agonists at the KOR without inducing β-arrestin2 recruitment. Recent reports point to the potential clinical value of opioids with reduced efficacy, partial agonists, and/or biased agonists, as effective and safer pain therapeutics.^15,69,97−99^

## Conclusion

In this study, we conducted a virtual screening campaign aiming for new, potent, selective, and nonbasic KOR agonists as potential candidates for therapeutic use as safer analgesics among others pathologies. We performed two 3D pharmacophore-based virtual screening methods in parallel and searched both NP libraries and synthetic compound libraries. Experimental evaluation of selected hit compounds revealed two ligands at the KOR, namely, **SalA-VS-07** and **SalA-VS-08**. Both compounds show affinity and potency at the KOR in the nanomolar range, with **SalA-VS-07** being a partial agonist at the KOR and **SalA-VS-08** being a full agonist at the KOR, together with a G protein-biased agonist profile. *In vitro* radioligand competition binding studies demonstrated both new ligands to be KOR selective. **SalA-VS-07** and **SalA-VS-08** share a common 2-methylspiro[6*H*-pyrazolo[1,5-*a*]pyrimidine-7,4′-piperidine]-5-one scaffold that was never reported as an opioid scaffold before. Altogether, our findings indicate that the applied KOR pharmacophore models and virtual screening workflows have a clear potential for the discovery of novel bioactive molecules at the KOR. The new chemotypes **SalA-VS-07** and **SalA-VS-08**, as NP mimicking compounds, represent a valuable starting point for chemical optimization toward the development of therapeutics for pain and other human disorders by selectively targeting the KOR.

## Experimental Section

### Virtual Screening

Two virtual screening methods were conducted in parallel. The first one is a classical 3D pharmacophore-based virtual screening performed in LigandScout,^53,100^ and the second one is a ROCS v3.4.3.0 screening implemented in OpenEye.^101,102^

### Query Generation for Virtual Screening

The 3D pharmacophore-based queries for virtual screening were obtained from the previously published binding hypothesis of SalA at the KOR.^52^ In particular, the 3D pharmacophore of this binding complex was generated in LigandScout v.4.4.3^53,100^ with an additional exclusion volume coat. As the 3D pharmacophore validation indicated that the omission of the C2-hydrogen bond acceptor (HBA) feature was meaningful, this feature was deleted to retrieve the final query 3D pharmacophore for the LigandScout screening. For the ROCS screening, SalA was extracted from the complex and loaded into the graphical user interface of ROCS v3.4.3.0^101,102^ of OpenEye. The automatically generated 3D pharmacophore features were manually corrected to resemble those of the initial binding complex. In particular, the HBA features of the furan and the C17-carbonyl group were deleted. Again, the HBA feature of the C2-acetoxy group was deleted after 3D pharmacophore validation to retrieve the final screening query.

### Query Validation

The virtual screening queries were evaluated for their ability to separate active molecules from decoys, and receiver operating characteristic (ROC) curves were generated for visualization of results. For this, databases of known actives and assumed inactives (decoys) were screened in an analogous way as later on the screening libraries of unknown activity. The active data set was generated from the ChEMBL database.^56^ In particular, all entries of the human KOR with any EC_50_ values measured in cell-based assays and a molecular weight of 300–500 Da were retrieved. Subsequently, all entries without exact EC_50_ values, missing EC_50_ values, or with units other than nM for their EC_50_ values were deleted. Only those entries measured in the [^35^S]GTPγS assays were kept. Entries uncharged at pH 7 were separated, and their duplicates deleted, leading to 82 uncharged KOR ligands with EC_50_ values from the pM range up to 5740 nM. Decoys of the active data set were generated by the DUD-E Web server,^57^ resulting in 4100 decoys. Thus, the active-decoy ratio is 1:50.

### Protein and Ligand Preparation

The human KOR model used for docking experiments is identical with those described in detail in the methods section of our previous publication.^52^ For MD simulations of momSalB, the respective cryo-EM structures were prepared as followed using MOE 2020.0901:^103^ The PDB-IDs 8DZP and 8DZQ were downloaded, and the intracellular bound G protein was deleted. Missing side chains were added, and broken loops were closed using the loop modeler panel implemented in MOE. Particularly, in 8DZP, the residues 203–205 in ECL2 were closed, while in 8DZQ, the residues 203–204 and 215–219 of ECL2 as well as 300–302 of ECL3 were modeled. The KOR wildtype sequence was restored by remutation of L135 to I135 according the UniProt-ID P41145.^104^ The protein geometry was optimized by solving Ramachandran plot outliers^105^ and atom clashes by careful side chain optimization and minimization using the OPLS-AA force field.^106^ The Protonate3D application^107^ implemented in MOE was used to assign ionization states at the pH of 7 and 300 K.

### Protein–Ligand Docking

Protein–ligand docking experiments were conducted to position the virtual screening hit compounds in the binding site of the active-state KOR X-ray crystal structure (PDB-ID: 6B73(24)) prepared as described above. All docking experiments were conducted in Gold v5.2.^108^ The KOR binding site was defined by a sphere of 15 Å radius around the terminal carbon atom (CD) of E209 and limited to a solvent accessible space. For each ligand, 15 separate and diverse docking hypothesis were generated; i.e., the root-mean-square deviation (RMSD) between the docking hypothesis must be ≥1.5 Å. During pose generation, pyramidal nitrogens were allowed to flip. The experiments were run with 100% search efficiency. Generated poses were scored by GoldScore.^109,110^ To promote binding modes with interactions indicated to be important by SAR studies, a pharmacophore constraint was applied. Poses with hydrogen bond acceptor (HBA) features at analogue positions as for SalA’s carbonyl groups at its C1-, C2-, and C4-substituents received higher scoring and were therefore favorably generated. These HBA features of SalA were indicated to be important in SAR studies.^42,52^ After docking pose generation, all poses were locally minimized in their protein environment by the MMFF94 force field implemented in LigandScout v.4.4.3.^53,100^ Gaussian shape similarity scores and 3D pharmacophore scores of docking poses were generated in LigandScout with the SalA binding mode as the reference pose.

### Molecular Dynamics (MD) Simulations

MD simulations were prepared in Maestro v2020^111^ and conducted using Desmond v2020-4.^112^ The protein–ligand complex models of momSalB (PDB-IDs: 8DZQ, 8DZP) were placed in a rectangular box with at least 10 Å distance between the receptor and the box edges and embedded in a POPC (1-palmitoyl-2-oleoylphosphatidylcholine) bilayer according to the OPM database^113^ entry of the active-state KOR (PDB-ID: 6B73). The remaining space was filled by TIP3P water^114^ and 1.5 M Na^+^ and Cl^–^ ions for isotonic conditions. For system parametrization, the Charmm36 force field was implemented into Maestro-setup using viparr-ffpublic.^115,116^ Five replicates of 200 ns each were performed per simulation system with 1000 sampled conformations per simulation run (in total 5000 per simulation system). The simulations were conducted with NPT ensemble conditions, i.e., with a constant number of particles, constant pressure (1.01325 bar), and constant temperature (300 K). After simulation, the protein was centered and the trajectories aligned according the backbone heavy atoms of the first conformation of the simulation using VMD v1.9.3.^117^ For MD simulation analysis, Dynophores were generated with the in-house developed Dynophore tool.^54,118,119^ Only interactions occurring for at least 5% of the simulation time were considered for evaluation. Of note, the MD simulations of SalA in complex with KOR were used from ref (52) with the same setting as described above.

### Drugs and Chemicals

Radioligands [^3^H]U69,593 (49.3 Ci/mmol), [^3^H]DAMGO (51.7 Ci/mmol), [^3^H]DPDPE (47.4 Ci/mmol), and [^35^S]GTPγS (1250 Ci/mmol) were purchased from PerkinElmer (Boston, MA, USA). Guanosine diphosphate (GDP), GTPγS, DAMGO, DPDPE, U69,593, nor-BNI, Salvinorin A, tris(hydroxymethyl) aminomethane (Tris), 2-[4-(2-hydroxyethyl)piperazin-1-yl]ethanesulfonic acid (HEPES), bovine serum albumin (BSA), and cell culture media and supplements were obtained from Sigma-Aldrich Chemicals (St. Louis, MO, USA). HS665 and HS378 were kindly provided by Helmut Schmidhammer (University of Innsbruck, Innsbruck, Austria). Morphine hydrochloride was obtained from Gatt-Koller GmbH (Innsbruck, Austria). **SalA-VS-1** to **SalA-VS-13** were obtained from AnalytiCon Discovery GmbH (Potsdam, Germany) and were prepared as 10 mM stock in DMSO and further diluted to working concentrations in the appropriate medium. All other chemicals were of analytical grade and obtained from standard commercial sources. All 13 tested compounds from the virtual screening campaign are >95% pure by HPLC analysis. HPLC data can be found in the Supporting Information (SI-section 04). Proton nuclear magnetic resonance (^1^H NMR) spectra and high resolution mass spectra (HRMS) for **SalA-VS-07** and **SalA-VS-08** are provided in the Supporting Information (SI-section 05).

### Cell Cultures and Membrane Preparation

CHO cells stably expressing the human opioid receptors (CHO-hKOR, CHO-hMOR, and CHO-hDOR) were kindly provided by Lawrence Toll (SRI International, Menlo Park, CA). CHO-hKOR cells were cultured in Dulbecco’s Modified Eagle’s Medium (DMEM) culture medium supplemented with 10% fetal bovine serum (FBS), 0.1% penicillin/streptomycin, 2 mM l-glutamine, and 0.4 mg/mL Geneticin (G418). CHO-hMOR and CHO-hDOR cells were cultured in DMEM/Ham’s F12 culture medium supplemented with 10% FBS, 0.1% penicillin/streptomycin, 2 mM l-glutamine, and 0.4 mg/mL Geneticin (G418). All cell cultures were grown at 37 °C under a humidified atmosphere of 95% air and 5% CO_2_. Membranes from CHO-hOR cells were prepared as previously described.^66^ Briefly, cells grown at confluence were removed from the culture plates by scraping, homogenized in 50 mM Tris-HCl buffer (pH 7.7) using a Dounce glass homogenizer, and then centrifuged once and washed by an additional centrifugation at 27,000*g* for 15 min at 4 °C. The final pellet was resuspended in 50 mM Tris-HCl buffer (pH 7.7) and stored at −80 °C until use. Protein content of cell membrane preparations was determined by the method of Bradford using BSA as the standard.^120^

### Radioligand Competitive Binding Assays for Opioid Receptors

Competitive binding assays were conducted on human opioid receptors stably transfected into CHO cells according to the published procedures.^66^ Binding assays were performed using [^3^H]U69,593 (1 nM), [^3^H]DAMGO (1 nM), or [^3^H]DPDPE (1 nM) for labeling KOR, MOR, or DOR, respectively. Nonspecific binding was determined using 10 μM of the unlabeled counterpart of each radioligand. Assays were performed in 50 mM Tris-HCl buffer (pH 7.4) in a final volume of 1 mL. Cell membranes (15–20 μg) were incubated with test compounds and the appropriate radioligand for 60 min at 25 °C. After incubation, reactions were terminated by rapid filtration through Whatman GF/C glass fiber filters. Filters were washed three times with 5 mL of ice-cold 50 mM Tris-HCl buffer (pH 7.4) using a Brandel M24R cell harvester (Gaithersburg, MD, USA). Radioactivity retained on the filters was counted by liquid scintillation counting using a Beckman Coulter LS6500 (Beckman Coulter Inc., Fullerton, CA, USA). All experiments were performed in duplicate and repeated at least three times with independently prepared samples.

### [^35^S]GTPγS Binding Assay for the KOR

Binding of [^35^S]GTPγS to membranes from CHO cells stably expressing the human KOR (CHO-hKOR) was conducted according to the published procedure.^66^ Cell membranes (10–15 μg) in Buffer A (20 mM HEPES, 10 mM MgCl_2_, and 100 mM NaCl, pH 7.4) were incubated with 0.05 nM [^35^S]GTPγS, 10 μM GDP, and test compounds in a final volume of 1 mL for 60 min at 25 °C. Nonspecific binding was determined using 10 μM GTPγS, and the basal binding was determined in the absence of test compound. For antagonist study, cell membranes were preincubated for 15 min with test ligand prior to the addition of 1 μM U69,593. Samples were filtered over Whatman GF/B glass fiber filters using a Brandel M24R cell harvester (Brandel, Gaithersburg, MD, USA). Radioactivity retained on the filters was counted by liquid scintillation counting using a Beckman Coulter LS6500 (Beckman Coulter Inc., Fullerton, CA, USA). All experiments were performed in duplicate and repeated at least three times with independently prepared samples.

### β-Arrestin2 Recruitment Assay for the KOR

The measurement of KOR-stimulated β-arrestin2 recruitment was performed using the DiscoveRx PathHunter eXpress β-arrestin2 assay (DiscoveRx, Birmingham, UK) according to the manufacturer’s protocol and published procedure.^68^ U2OS cells stably coexpressing the human KOR and the enzyme acceptor-tagged β-arrestin2 fusion protein (U2OS–hKOR−β-arrestin2 cells) were seeded in cell plating medium into 384-well white plates (Greiner Bio-One, Austria) at a density of 2,000 cells in 20 μL per well and maintained for 48 h at 37 °C. After incubation with various concentrations of test compounds in PBS for 180 min at 37 °C, the detection mix was added, and incubation was continued for an additional 60 min at room temperature. Chemiluminescence was measured with a Varioskan LUX Multimode Microplate Reader (ThermoFischer Scientific Inc., USA). All experiments were performed in duplicate and repeated at least three times with independently prepared samples.

### Data and Statistical Analysis

Experimental data were graphically processed and statistically analyzed using GraphPad Prism Software (GraphPad Prism Software Inc., San Diego, CA). In *in vitro* binding assays, inhibition constant (*K*_i_, nM), potency (EC_50_, nM), and efficacy (*E*_max_,_%_) values were determined from concentration–response curves by nonlinear regression analysis. The *K*_i_ values were determined by the method of Cheng and Prusoff.^121^ In the [^35^S]GTPγS binding assays, efficacy was determined relative to the reference KOR full agonist, U69,593. All data are presented as the mean ± SEM.

## Supporting Information Available

(1) Chemical structures of reference opioid receptor ligands used for pharmacological characterization (Figure S1), (2) chemical space analysis of virtual screening hits (Figures S2 and S3), (3) chemical structures of known natural products with opioid receptor activity (Figure S4), (4) purity data for inactive virtual screening hits, and (5) analytical characterization of active compounds **SalA-VS-07** and **SalA-VS-8**: NMR, high resolution MS spectra, and HPLC chromatograms (PDF)
KOR in modeled complex between **SalA-VS07** (PDB)
KOR in modeled complex between **SalA-VS08** (PDB)
Molecular Formula Strings (CSV)

## Supplementary Material

jm4c00590_si_001.pdf

jm4c00590_si_002.pdb

jm4c00590_si_003.pdb

## Abbreviations Used

AUC: area under the curve
CHO: Chinese hamster ovary
DAMGO: [d-Ala^2^,*N*-Me-Phe^4^, Gly-ol^5^]enkephalin
DOR: delta-opioid receptors
DPDPE: [d-Pen^2^,d-Pen^5^]enkephalin
ER: estrogen receptor
GPCRs: G protein-coupled receptors
HBA: hydrogen bond acceptor feature
HY: hydrophobic contact feature
KOR: kappa-opioid receptor
MD: molecular dynamics
MOR: mu-opioid receptor
NOP: nociceptin/orphanin FQ peptide
NP: natural product
PDB: protein database
RMSD: root-mean-square deviation
ROC: receiver operating characteristic
SalA: Salvinorin A
SalB: Salvinorin B
[^35^S]GTPγS: guanosine-5′-*O*-(3-[^35^S]thio)-triphosphate
U69,593: *N*-methyl-2-phenyl-*N*-[(5*R*,7*S*,8*S*)-7-(pyrrolidin-1-yl)-1-oxaspiro[4.5]dec-8-yl]acetamide
