# Supplementary material for: Discovery of Novel, Selective, and Nonbasic Agonists for the Kappa-Opioid Receptor Determined by Salvinorin A-Based Virtual Screening
Source: J Med Chem. 2024 Aug 1;67(16):13788–801. doi: 10.1021/acs.jmedchem.4c00590 (PMC11345774; doi:10.1021/acs.jmedchem.4c00590)
Supplement: Supplementary file 1 — jm4c00590_si_001.pdf [file jm4c00590_si_001.pdf]

Discovery of Novel, Selective, and Nonbasic Agonists for the Kappa-Opioid Receptor Determined by Salvinorin A-Based Virtual Screening

Kristina Puls[1], Aina-Leonor Olivé-Marti[2], Siriwat Hongnak[2], David Lamp[2], Mariana Spetea[2]* and Gerhard Wolber[1]*

[1]Department of Pharmaceutical Chemistry, Institute of Pharmacy, Freie Universität Berlin, Königin-Luise-Str. 2-4, 14195 Berlin, Germany; kristina.puls@fu-berlin.de (K.P.) gerhard.wolber@fu-berlin.de

[2]Department of Pharmaceutical Chemistry, Institute of Pharmacy and Center for Molecular Biosciences Innsbruck (CMBI), University of Innsbruck, Innrain 80-82, 6020 Innsbruck, Austria; aolive.marti@gmail.com (A.-L.O.-M) siriwat.hongnak,@uibk.ac.at (S.H.) davidlamp42@gmail.com (D.L.) mariana.spetea@uibk.ac.at (M.S.)

*Correspondence: gerhard.wolber@fu-berlin.de (G.W.); mariana.spetea@uibk.ac.at (M.S.); Tel.: +49-30-838-52686 (G.W.); +43-512-507-58277 (M.S.)

Table of Contents

1. Chemical Structures of Reference Ligands (Figure S1). 2

2. Chemical Space of Virtual Screening Hits (Figure S2 & S3) 3

3. Natural Products with Opioid Activity (Figure S4) 5

4. HPLC Traces for Inactive Virtual Screening Hits 6

5. Analytical Characterization of Active Compounds SalA-VS-07 and SalA-VS-08 12

^{1}H NMR: SalA-VS-07 13

HRMS SalA-VS-07 13

HPLC trace of SalA-VS-07 14

^{1}H NMR SalA-VS-08 15

HRMS SalA-VS-08 15

HPLC trace of SalA-VS-08 16

1. Chemical Structures of Reference Ligands (Figure S1).

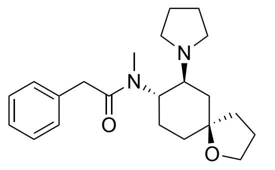

U69593

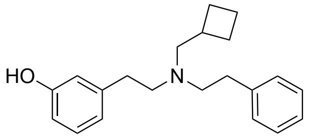

HS665

H-Tyr-d-Ala-Gly-N-MePhe-Gly-ol
DAMGO

H-Tyr-D-Pen(1)-Gly-Phe-D-Pen(1)-OH
DPDPE

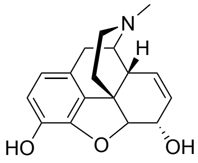

Morphine

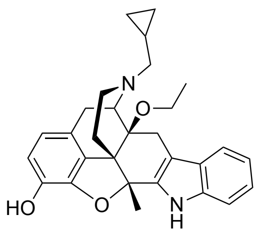

HS378

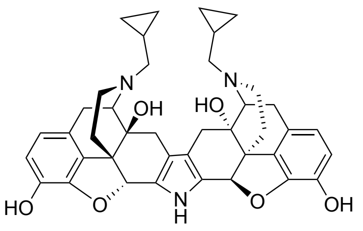

Nor-BNI

Figure S1. Chemical structures of reference opioid receptor ligands used for pharmacological characterization in this study

2. Chemical Space of Virtual Screening Hits (Figure S2 & S3)

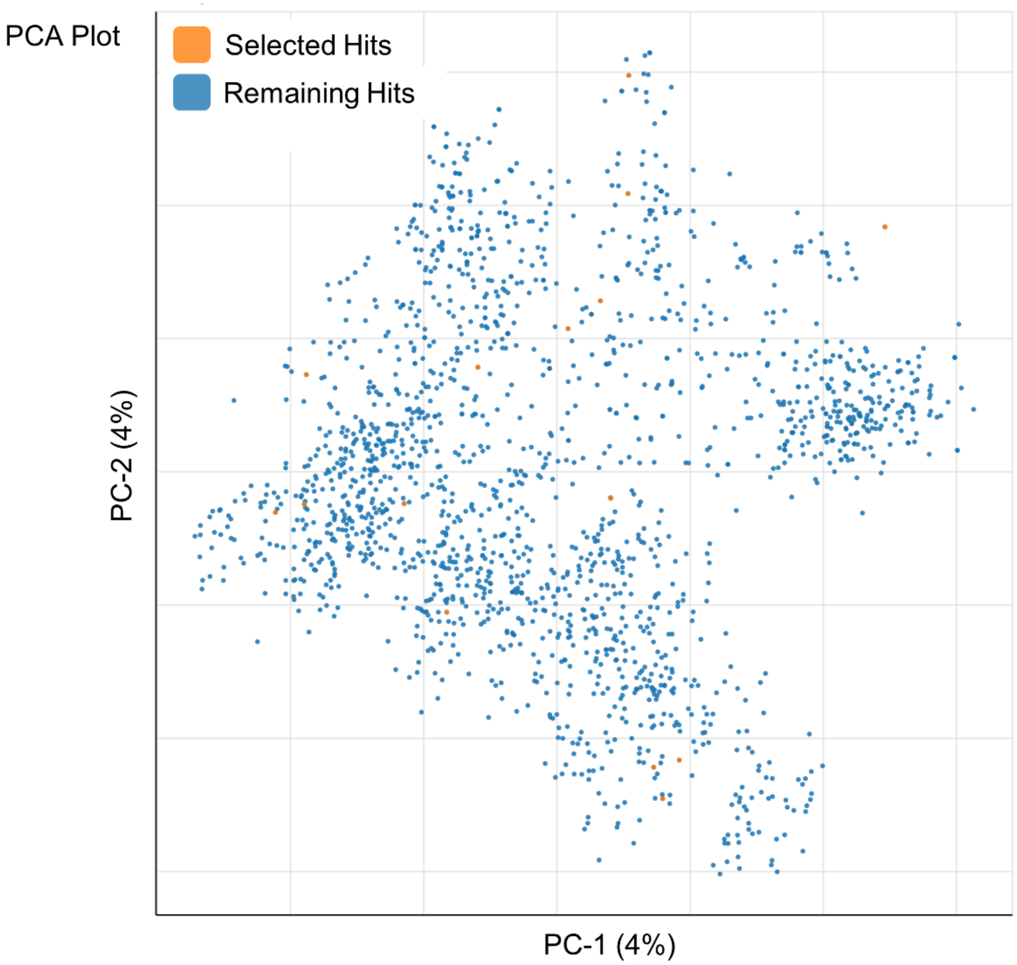

Figure S2. Principal component analysis (PCA) of the chemical space of virtual screening hits. Compounds selected for pharmacological experiments are highlighted in orange while the remaining hit molecules are visualized in blue. Plot was generated with ChemPlot[1] structural analysis.

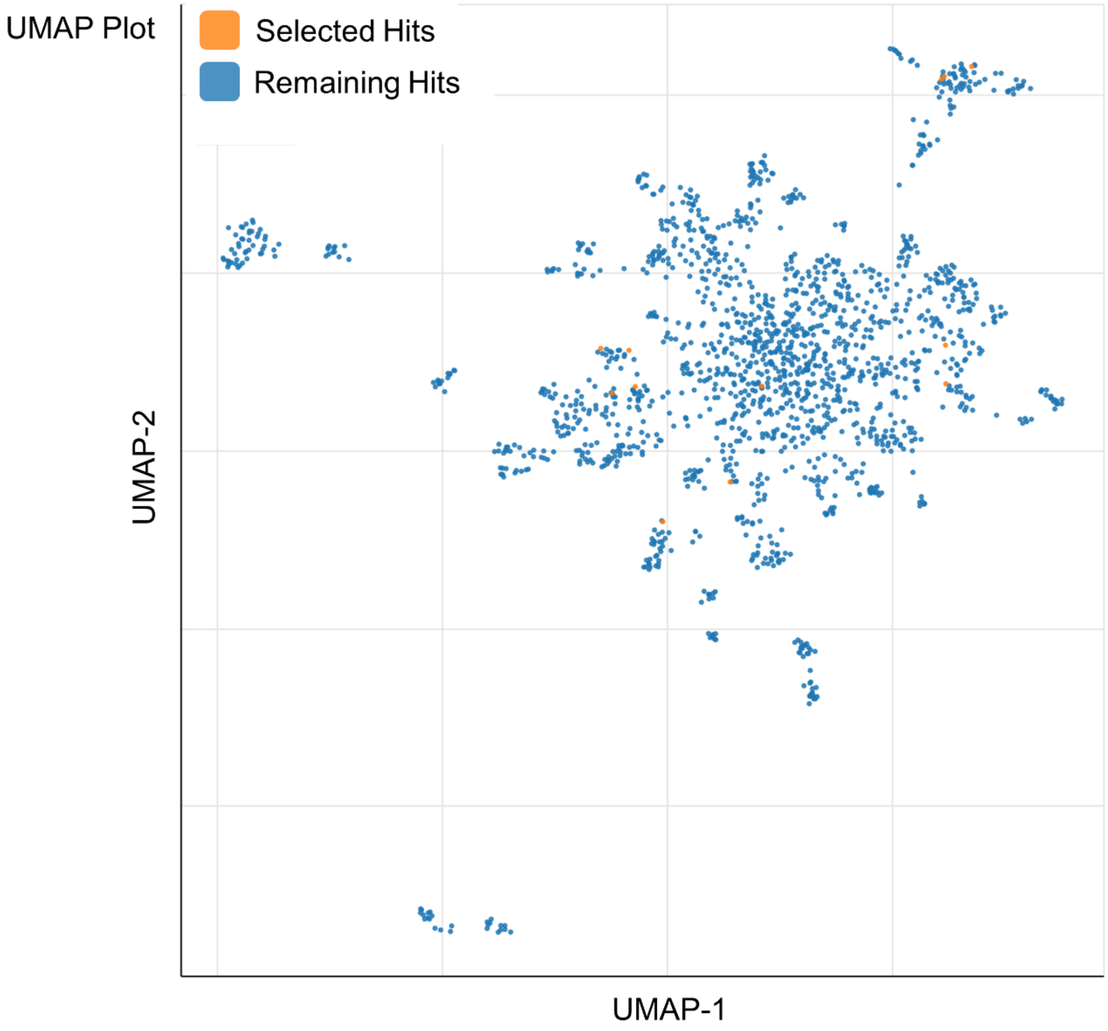

Figure S3. Visualization of the chemical space of virtual screening hits by non-linear Uniform Manifold Approximation and Projection (UMAP) dimensionality reduction. Compounds selected for pharmacological experiments are highlighted in orange while the remaining hit molecules are visualized in blue. Plot was generated with ChemPlot structural analysis. (Cihan Sorkun, M.; Mullaj, D.; Koelman, J. V. A.; Er, S. ChemPlot, a Python library for chemical space visualization. *Chemistry-Methods* 2022, 2, e202200005. DOI: 10.1002/cmtd.202200005)

3. Natural Products with Opioid Activity (Figure S4)

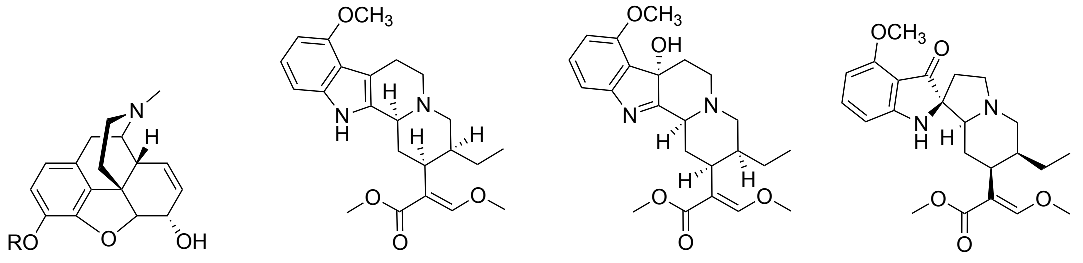

**Morphine** (R=H)<br>**Codeine** (R=CH3)	**Mitragynine**	**7-Hydroxy-Mitragynine**	**Mitragynine pseudoindoxyl**

**Rubiscolin-5**	YPLDL
**Rubiscolin-6**	YPLDLF
**Dynorphin A1-13**	YGGFLRRIRPKLK
**Met-Enkephalin**	**YGGF**M
**β-Endorphin**	**YGGF**MTSEKSQTPLVTLFKNAIIKNAYKKGE
**Nociceptin**	**FGGF**TGARKSARKLANQ

Figure S4. Chemical structures of known natural products with opioid receptors activity. Morphine and codeine are shown as representatives for morphinan-based natural products. Dynorphin A, met-enkephalin, β-endorphin, and nociceptin were chosen as representatives of endogenous opioid peptides. The conserved opioid peptide motifs YGGF/FGGF are highlighted in bold.

4. HPLC Traces for Inactive Virtual Screening Hits

All compounds are >95% pure by HPLC analysis. As per guidelines of the Journal of Medicinal Chemistry, HPLC traces are included below. This section contains HPLC traces for the inactive compounds. High-resolution mass, NMR characterization and HPLC traces of active compounds SalA-VS-07 and SalA-VS-08 are presented in section 5.

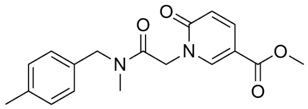

SalA-VS-01

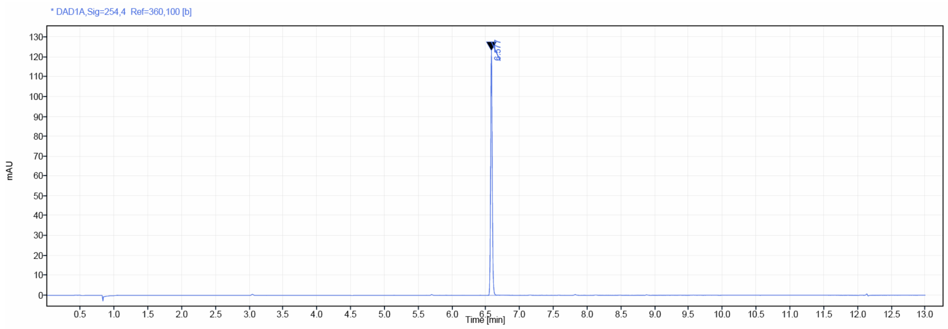

Signal: * DAD1A,Sig=254,4 Ref=360,100 [b]

RT [min]	Type	Width [min]	Area	Height	Area%	Name
6.577	BV	0.18	205.15	123.19	100.00	
		**Sum**	**205.15**			

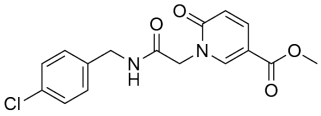

SalA-VS-02

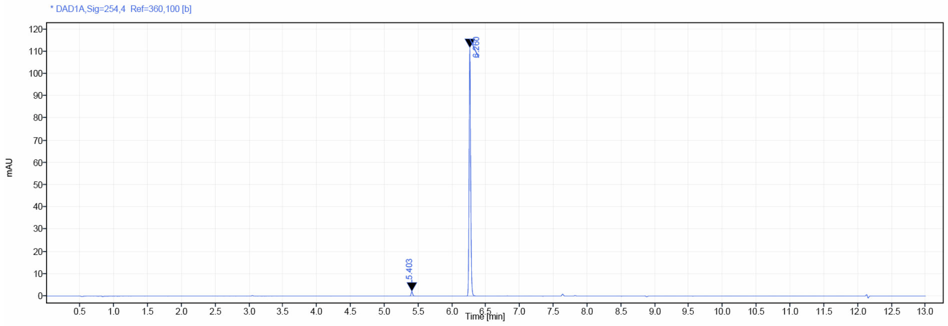

Signal: * DAD1A,Sig=254,4 Ref=360,100 [b]

RT [min]	Type	Width [min]	Area	Height	Area%	Name
5.403	BV	0.11	3.29	2.02	1.80	
6.260	BV	0.19	179.24	111.56	98.20	
		**Sum**	**182.53**			

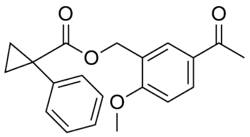

SalA-VS-03

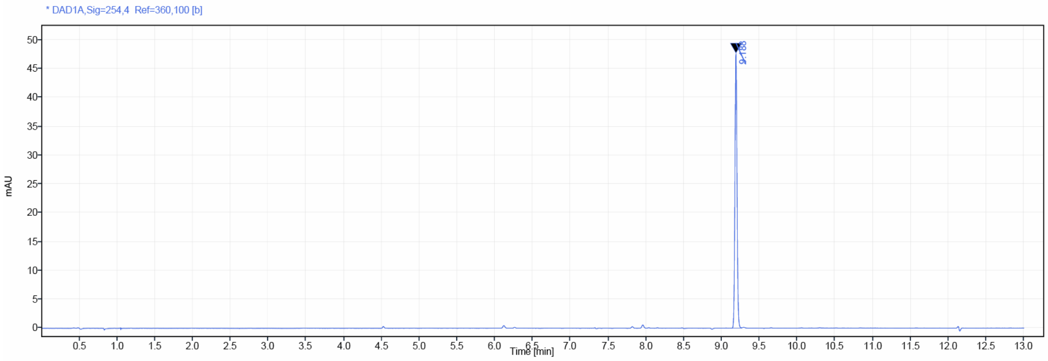

Signal: * DAD1A,Sig=254,4 Ref=360,100 [b]

RT [min]	Type	Width [min]	Area	Height	Area%	Name
9.188	BV	0.15	92.18	47.67	100.00	
		**Sum**	**92.18**			

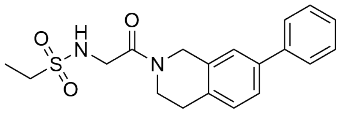

SalA-VS-04

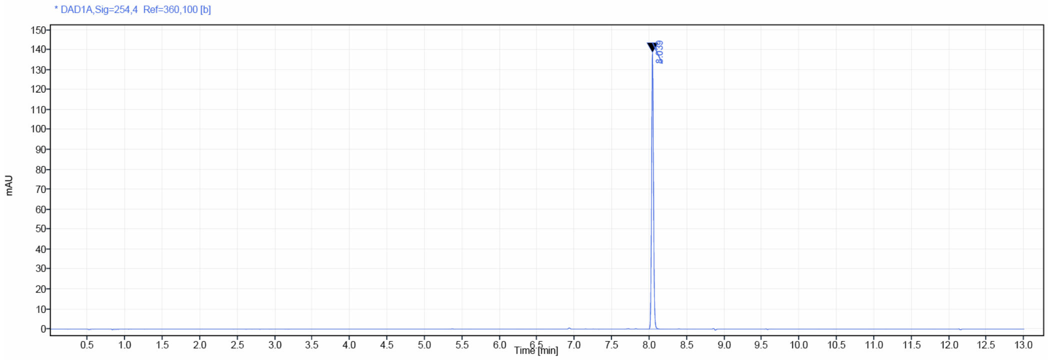

Signal: * DAD1A,Sig=254,4 Ref=360,100 [b]

RT [min]	Type	Width [min]	Area	Height	Area%	Name
8.039	BV	0.25	246.49	138.55	100.00	
		**Sum**	**246.49**			

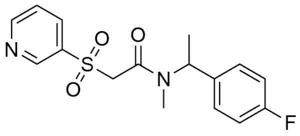

SalA-VS-05

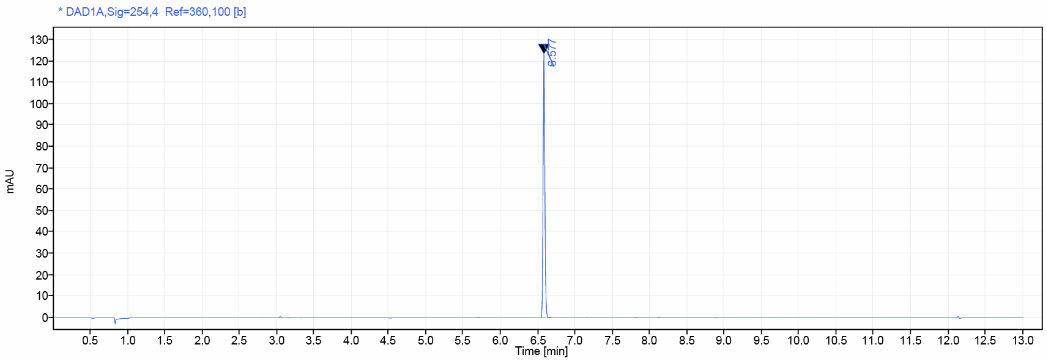

Signal:	* DAD1A,Sig=254,4 Ref=360,100 [b]					
**RT [min]**	**Type**	**Width [min]**	**Area**	**Height**	**Area%**	**Name**
6.577	BV	0.18	205.15	123.19	100.00	
		**Sum**	**205.15**			

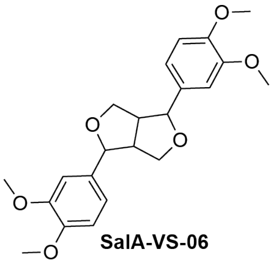

SalA-VS-06

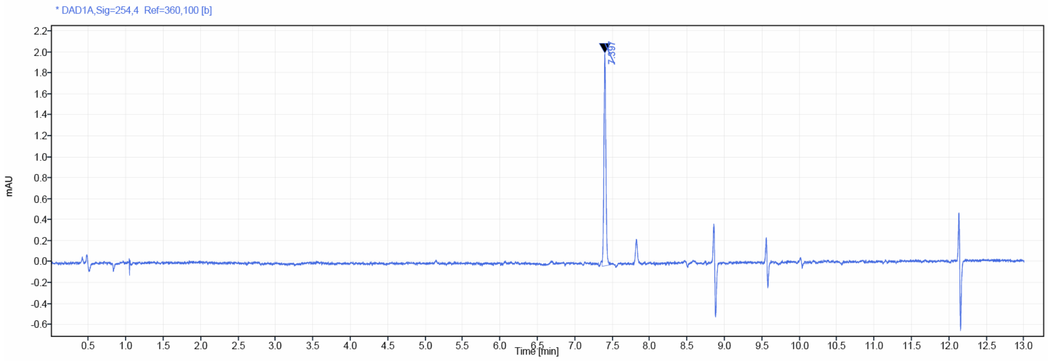

Signal:	* DAD1A,Sig=254,4 Ref=360,100 [b]					
**RT [min]**	**Type**	**Width [min]**	**Area**	**Height**	**Area%**	**Name**
7.397	VB	0.11	3.72	2.02	100.00	
		**Sum**	**3.72**			

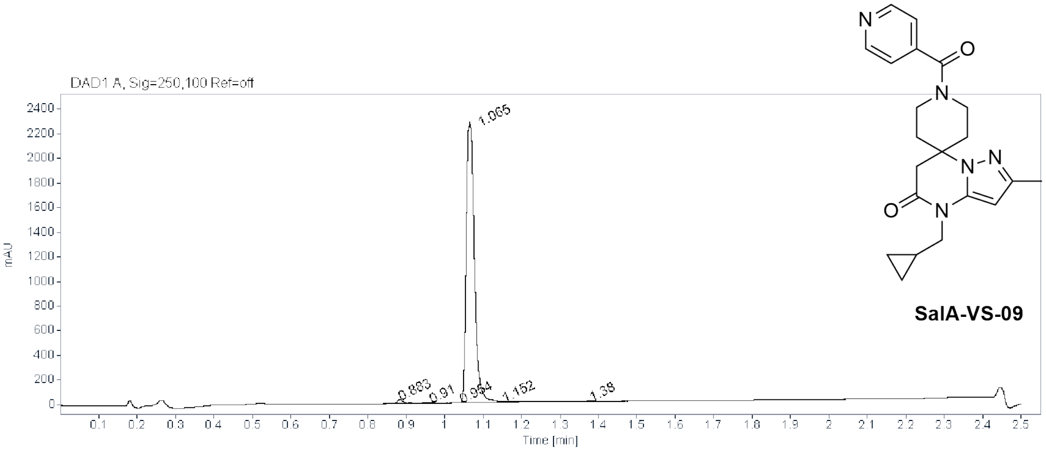

SalA-VS-09

Signal: DAD1 A, Sig=250,100 Ref=off

**RT [min]**	Peak Area Percent
0.883	0.91
0.910	0.12
0.954	0.25
1.065	98.13
1.152	0.35

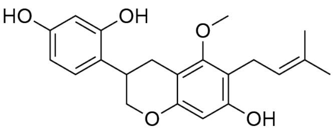

SalA-VS-10

ELSD

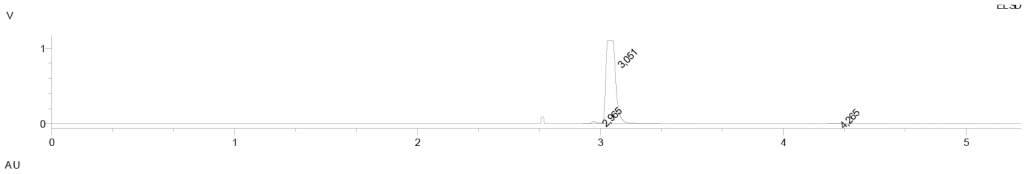

Peak Table C-3357-H-D11

AD2

Peak#	Ret. Time	Area	Area%
1	2,965	61715	1,507
2	3,051	4029419	98,408
3	4,265	3457	0,084
Total		4094590	100,000

PDA Ch1 215nm

Peak#	Ret. Time	Area	Area%
Total			

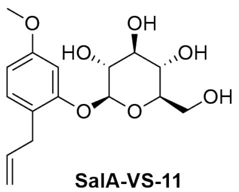

SalA-VS-11

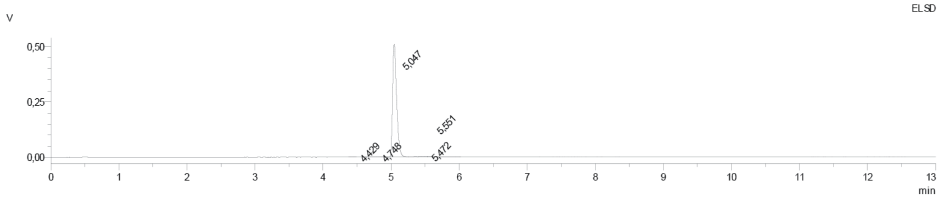

Peak Table MEGx_Box_0408_G11_210121
AD2

Peak#	Ret. Time	Area	Area%
1	4,429	3387	0,153
2	4,748	10533	0,475
3	5,047	2125897	95,919
4	5,472	40315	1,819
5	5,551	36204	1,633
Total		2216336	100,000

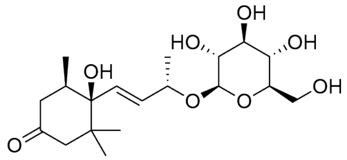

SalA-VS-12

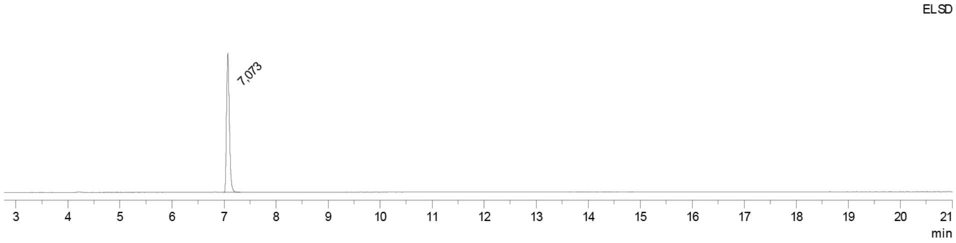

Peak Table MEGx_Store_MTP-1577_F12_190313
AD2

Peak#	Ret. Time	Area	Area%
1	7,073	1123968	100,000
Total		1123968	100,000

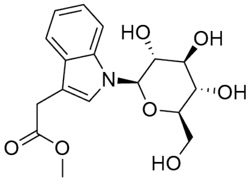

SalA-VS-13

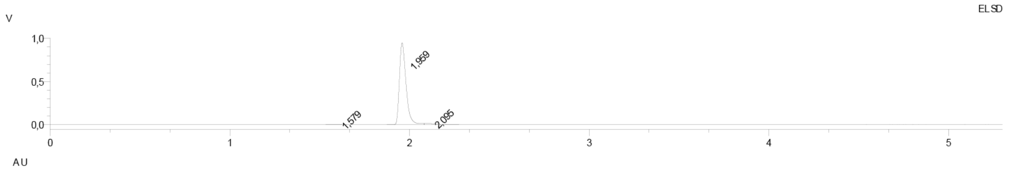

Peak Table H-2192-G-D08
AD2

Peak#	Ret. Time	Area	Area%
1	1,579	4420	0,176
2	1,959	2442569	97,336
3	2,095	62420	2,487
Total		2509409	100,000

PDA Ch1 215nm

Peak#	Ret. Time	Area	Area%
Total			

5. Analytical Characterization of Active Compounds SalA-VS-07 and SalA-VS-08

NMR: Proton nuclear magnetic resonance (^1H NMR) spectra were recorded at 400 MHz on a Bruker Avance 4 Neo spectrometer; chemical shifts were calibrated using residual nondeuterated solvents $CHCl_3$ (δ = 7.26 ppm) or MeOH (δ = 3.31 ppm) and expressed in δ ppm. Coupling constants (*J*) are reported in hertz (Hz). Multiplicities are given as singlet (s), doublet (d), triplet (t), multiplet (m), and broad (br).

High resolution mass spectra (HRMS) were obtained on a Thermo Scientific Q Executive, ESI source, and positive ion-mode.

^{1}H NMR: SalA-VS-07

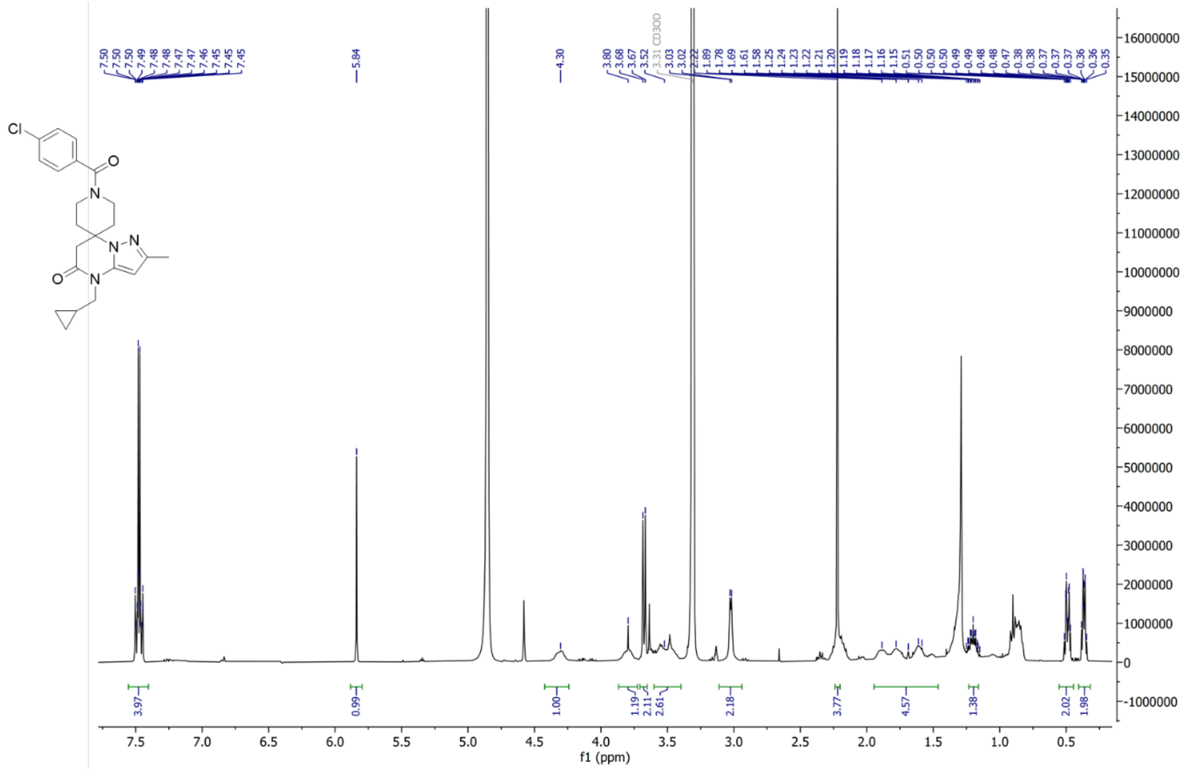

1-(4-chlorobenzoyl)-4'-(cyclopropylmethyl)-2'-methyl-4'*H*-spiro[piperidine-4,7'-pyrazolo[1,5-*a*]pyrimidin]-5'(6'*H*)-one (SalA-VS-07)

^{1}H NMR (400 MHz, MeOD) δ 7.50 – 7.45 (m, 4H), 5.84 (s, 1H), 4.30 (br. s, 1H), 3.80 (br. s, 1H), 3.68 (d, *J* = 7.1 Hz, 2H), 3.52 (br. s, 2H), 3.03 (d, *J* = 4.8 Hz, 2H), 2.22 (s, 3H), 1.69 (br, 4H), 1.25 – 1.15 (m, 1H), 0.51 – 0.47 (m, 2H), 0.38 – 0.35 (m, 2H).

HRMS (ESI$^+$): m/z calcd. for $C_{22}H_{26}N_4O_2Cl$ [M + H]$^+$, 413.1739; found, 413.1749.

HRMS SalA-VS-07

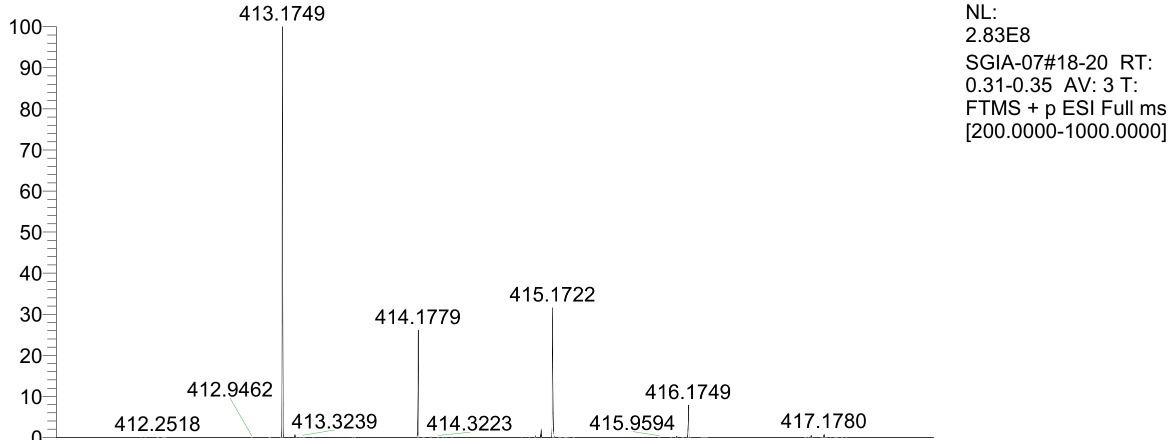

HPLC trace of SalA-VS-07

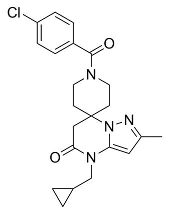

SalA-VS-07

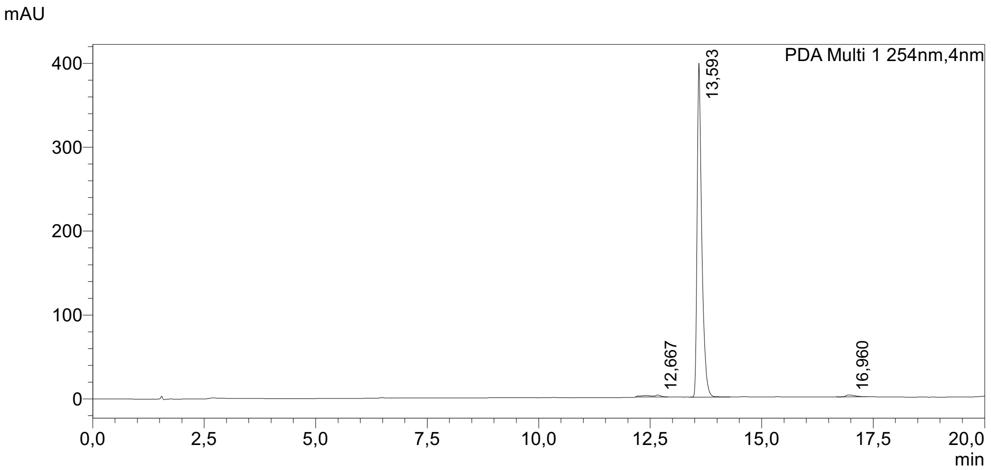

<Peak Table>

PDA Ch1 254nm

Peak#	Ret. Time	Area	Mark	Height	Area%
1	12,667	51629	M	2324	1,712
2	13,593	2932203	M	397995	97,219
3	16,960	32257	M	2288	1,070
Total		3016090		402607	100,000

^{1}H NMR SalA-VS-08

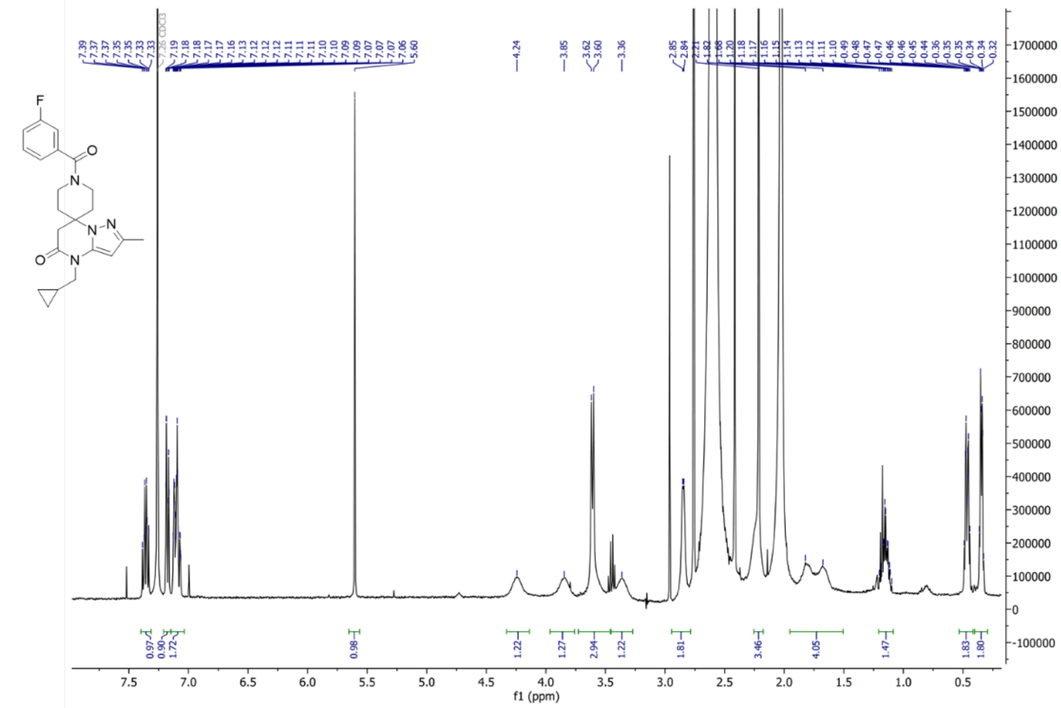

4'-(cyclopropylmethyl)-1-(3-fluorobenzoyl)-2'-methyl-4'*H*-spiro[piperidine-4,7'-pyrazolo[1,5-*a*]pyrimidin]-5'(6'*H*)-one (SalA-VS-08)

^{1}H NMR (400 MHz, $CDCl_3$) δ 7.39 – 7.33 (m, 1H), 7.17 (dt, *J* = 7.6, 1.2 Hz, 1H), 7.13 – 7.06 (m, 2H), 5.60 (s, 1H), 4.24 (br. s, 1H), 3.85 (br. s, 1H), 3.61 (d, *J* = 7.1 Hz, 3H), 3.36 (br. s, 1H), 2.85 (d, *J* = 3.8 Hz, 3H), 2.21 (s, 3H), 1.74 (br. d, 4H), 1.20 – 1.10 (m, 1H), 0.49 – 0.44 (m, 2H), 0.36 – 0.32 (m, 2H).

HRMS (ESI^+): m/z calcd. for $C_{22}H_{26}N_4O_2F$ $[M + H]^+$, 397.2034; found, 397.2026.

HRMS SalA-VS-08

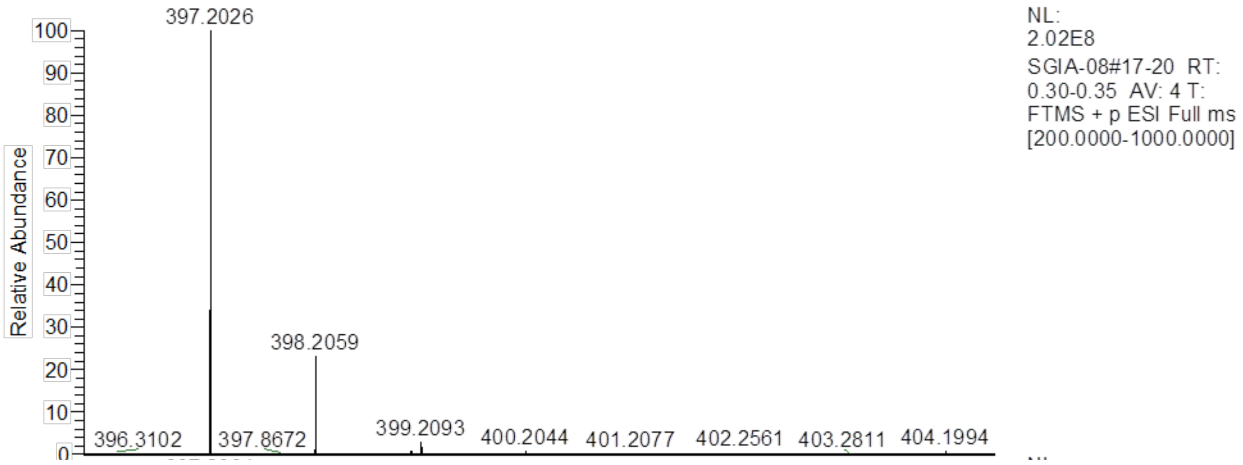

HPLC trace of SalA-VS-08

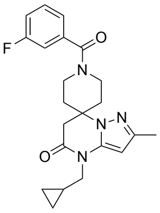

SalA-VS-08

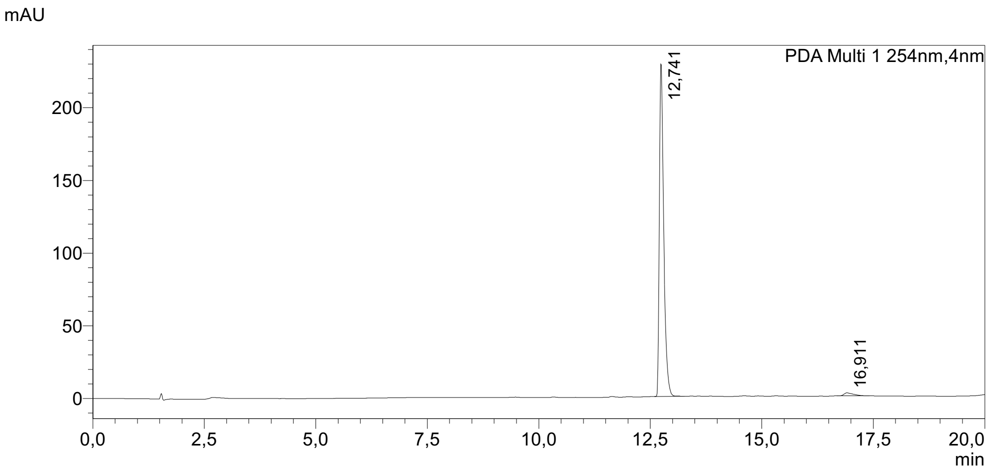

<Peak Table>

PDA Ch1 254nm

Peak#	Ret. Time	Area	Mark	Height	Area%
1	12,741	1540259	M	228656	98,011
2	16,911	31257	M	2094	1,989
Total		1571516		230749	100,000
